# Employing MIC Data for Mink Pathogens to Propose Tentative Epidemiological Cut-Off Values: A Step Toward Rationalizing Antimicrobial Use in Mink

**DOI:** 10.3389/fvets.2020.544594

**Published:** 2020-10-21

**Authors:** Nanett Kvist Nikolaisen, Amir Atabak Ronaghinia, Desiree Corvera Kløve Lassen, Chaza Nazih Chehabi, Mikkel Lindegaard, Tina Struve, Mariann Chriél, Peter Damborg, Gunnar Kahlmeter, Lars Bogø Jensen, Karl Pedersen

**Affiliations:** ^1^National Food Institute, Research Group for Microbiology and Hygiene, Technical University of Denmark, Kongens Lyngby, Denmark; ^2^Department of Health and Diagnostics, Kopenhagen Fur A.M.B.A., Glostrup, Denmark; ^3^Department of Veterinary and Animal Sciences, Faculty of Health and Medical Sciences, University of Copenhagen, Copenhagen, Denmark; ^4^Centre for Diagnostics, Technical University of Denmark, Kongens Lyngby, Denmark; ^5^Klinisk Mikrobiologi, Växjö, Sweden; ^6^National Veterinary Institute, Uppsala, Sweden

**Keywords:** ECOFF, MIC, pharmacodynamics, mink, *E. coli*, *P. aeruginosa*, *S. canis*, *S. delphini*

## Abstract

Optimizing antimicrobial dosage regimens and development of breakpoints for antimicrobial susceptibility testing are important prerequisites for rational antimicrobial use. The objectives of the study were (1) to produce MIC data for four mink pathogens and (2) to employ these MIC data to support the development of tentative epidemiological cut-off values (TECOFFs), which may be used for future development of mink-specific antimicrobial dosages and breakpoints. Broth microdilution was used to establish MIC distributions for 322 mink bacterial isolates of clinical origin from six European mink-producing countries. The included species were *E. coli* (*n* = 162), *S. delphini* (*n* = 63), *S. canis* (*n* = 42), and *P. aeruginosa* (*n* = 55). Sixty-four *E. coli* isolates and 34 *S. delphini* isolates were whole-genome sequenced and analyzed for antimicrobial resistance genes. No EUCAST MIC data are available on *S. delphini* and *S. canis*, hence tentative ECOFFs were suggested for the majority of the tested antimicrobials. For *E. coli* and *P. aeruginosa*, the wildtype distributions were in accordance with EUCAST data. Overall, the genotypes of the sequenced isolates were in concordance with the phenotypes. These data constitute an important piece in the puzzle of developing antimicrobial dosages and clinical breakpoints for mink. Until pharmacokinetic and clinical data become available, the (tentative) ECOFFs can be used for monitoring resistance development and as surrogates for clinical breakpoints.

## Introduction

As in other species, mink become clinically ill due to various infectious agents, including a range of bacterial pathogens causing decreased animal welfare and affecting commercial fur production. Common bacterial pathogens in mink include *Escherichia coli*, which may cause diarrhea, *Pseudomonas aeruginosa*, which may cause hemorrhagic pneumonia, *Staphylococcus delphini*, which may cause urinary tract infections, and *Streptococcus canis*, which may cause skin infections (1). Bacterial infections in mink often require antimicrobial treatment. However, antimicrobial therapy in the mink industry is mostly based on empirical knowledge since clinical breakpoints and antimicrobial dosage regimens for mink are unavailable. Such non-evidence-based practice might lead to treatment failure, toxicity, and/or selection for antimicrobial resistance. Optimal treatment of bacterial infections relies on pharmacodynamic data pertaining to bacterial target pathogens and antimicrobial agents, respectively. Exploiting such data for development of clinical breakpoints and dosage regimens can help ensure a proper drug choice and an adequate antimicrobial concentration at the site of infection.

The European Committee on Antimicrobial Susceptibility Testing (EUCAST) is a scientific committee focusing on antimicrobial resistance and providing guidelines for procedures and interpretation of antimicrobial susceptibility testing. EUCAST defines the wildtype as isolates that have not acquired phenotypically detectable resistance mechanisms, and the epidemiological cut-off value (ECOFF) as the highest minimum inhibitory concentration (MIC) for the wildtype population (2). Thus, ECOFFs distinguish between isolates with and without phenotypically identifiable antimicrobial resistance, non-wildtype and wildtype, respectively. Noteworthy, ECOFFs cannot necessarily be used to predict the outcome of therapy. Using the ECOFF as a biological phenomenon, *in vitro* resistance can be measured and the development of resistance can be monitored despite the lack of clinical breakpoints (3–6).

Several requirements need to be met to suggest an ECOFF, e.g., the dataset needs to include at least five MIC distribution generated from separate laboratories. Furthermore, at least 15 isolates per MIC distribution must be represented in the putative wildtype population, and only a single peak (mode) in the MIC distribution of the putative wildtype distribution is allowed. One of the requirements for the aggregated distribution is that there must be at least 100 MIC values in the putative wildtype distribution (2). If some requirements are not met, a tentative ECOFF (TECOFF) can be proposed until more data become available (2).

Several antimicrobials can be used in veterinary practice. However, some are also applied in human medicine and for the treatment of infections involving multi-drug resistant bacteria. The World Health Organization (WHO) has published Model List of Essential Medicines 2019 (7). One of the included antimicrobials is marked as reserved (colistin), five are marked as accessible [amoxicillin, doxycycline, spectinomycin, trimethoprim and sulfamethoxazole in combination with trimethoprim (SXT)], and one antimicrobial (lincomycin) as “watch.” Tylosin is only licensed for use in animals.

In this study, 322 bacterial isolates representing four bacterial species were tested against eight antimicrobials using an extended range of concentrations. Results of the relevant antimicrobials for each bacterial species are included (2–7 antimicrobials per species). The majority of the resulting MIC distributions allowed us to identify the wildtype and non-wildtype populations. This study provides valuable information on *in vitro* antimicrobial resistance in clinical bacteria from mink. Additionally, the MIC distributions data and (T)ECOFFs are important tools, together with pharmacokinetic and clinical data, for constructing dosage regimens and for suggesting relevant breakpoints.

## Materials and Methods

### Bacterial Isolates

Bacterial isolates were obtained from clinical material from mink submitted to diagnostic laboratories (The National Veterinary Institute DTU, Lyngby, Denmark; Institute for Experimental Pathology, Reykjavík, University of Iceland; veterinary clinic Pecon BV, Gemert, the Netherlands; INVESAGA Group, Department of Animal Pathology, University of Santiago de Compostela, Lugo, Spain; Finnish Food Authority, Seinäjoki, Finland) in the period 2006–2018. Each submission to the laboratory could consist of more than one animal. A maximum of one isolate of each of the four bacterial species was collected from each submission. A farm could be represented more than once if samples were submitted to the laboratory repeatedly for investigation. There was no limitation as to how many times each farm could be represented over the 12-year sampling period. Also, the antimicrobial treatment history for the farms was not a criterion for the inclusion of bacterial isolates. The mink industry follows the same seasonal pattern all over the world, and the animals have been submitted from the beginning of whelping (April) until pelting (November). The following species were included in the study: *E. coli* (*n* = 162), *S. delphini* (*n* = 63), *S. canis* (*n* = 42), and *P. aeruginosa* (*n* = 55). Isolates originated from Denmark, Finland, Iceland, Lithuania, the Netherlands, and Spain (Table 1). All isolates included in this study were identified by MALDI-TOF as described in Nikolaisen et al. (8).

**Table 1:** The 322 isolates included in the study, divided into species and country of origin.

	**Denmark**	**Iceland**	**The Netherlands**	**Finland**	**Spain**	**Lithuania**	**Total**
*Escherichia coli*	103	23	4	26	5	1	162
*Staphylococcus delphini*	24	14	1	20	4		63
*Pseudomonas aeruginosa*	24	13	18				55
*Streptococcus canis*	35	1	6		1		42

### Antimicrobial Susceptibility Testing

All isolates were investigated using the broth microdilution semiautomated technique Sensititre (ThermoFisher Scientific, UK) according to methods described by the Clinical and Laboratory Standards Institute (9). For *E. coli, S. delphini*, and *P. aeruginosa*, cation-adjusted Mueller-Hinton broth (CAMHB) was used, and panels were incubated at 35 ±2°C for 16–20 h, whereas for *S. canis* CAMHB with lyzed horse blood was used and panels were incubated at 35 ± 2°C for 20–24 h (9, 10). Based on data from the national veterinary prescription database VetStat (11, 12), some of the most frequently used antimicrobials in mink production in Denmark were chosen for designing a custom-made panel. This panel contained 2-fold dilutions of amoxicillin (range 0.25–512 μg/mL), colistin (0.06–128 μg/mL), spectinomycin (0.25–512 μg/mL), sulfamethoxazole-trimethoprim 19:1 (0.03–64 μg/mL), doxycycline (0.06–128 μg/mL), lincomycin (0.06–128 μg/mL), sulfamethoxazole (0.5–512 μg/mL), and tylosin (0.12–128 μg/mL). Antimicrobial concentration ranges were based on MIC distributions in the EUCAST MIC database (13) and earlier reports on prevalence of antimicrobial resistance in bacterial pathogens from mink (1, 8). A subset of isolates was further tested for susceptibility to trimethoprim (*E. coli*: *n* = 53, *S. delphini*: *n* = 38, *S. canis*: *n* = 26) and penicillin (*S. delphini*: *n* = 18) (Supplementary Tables 1, 2) by broth microdilution (9). Trimethoprim test was performed to determine the added effect of the combinational drug sulfamethoxazole and trimethoprim. Susceptibility to penicillin was tested in isolates harboring the *blaZ* gene. *Escherichia coli* ATCC 25922, *Staphylococcus aureus* ATCC 29213, and *Pseudomonas aeruginosa* ATCC 27853 were used as quality control strains. Every 10th Sensititre panel was inspected and evaluated by a second investigator.

### Epidemiological Cut-Off Values

The protocol for data collection was performed according to the EUCAST SOP 10.1 for ECOFF setting (2). The MICs were determined in three different laboratories by different investigators at (1) the National Food Institute at the Technical University of Denmark, (2) the Department of Veterinary and Animal Sciences at the University of Copenhagen, and (3) the Institute for Experimental Pathology at the University of Iceland.

Firstly, the MIC distributions were visually inspected (e.g., the Gaussian wildtype MIC distributions were identified) to ascertain that the “ECOFFinder” version 2.0 software could be applied (nonlinear regression, at 99%) (4). Additionally, the MIC distributions for each antimicrobial agent and species were compared to the modes and ECOFFs already established and available in the EUCAST database (13).

Prior to analyzing results for SXT, MIC distributions for sulfamethoxazole and trimethoprim were created separately. A “true” SXT wildtype MIC distribution was solely defined on organisms, which were independently wildtype to both agents. Isolates in the SXT wildtype population with MICs > ECOFF for sulfamethoxazole alone were omitted, as the effect of the combinational drug, SXT, must be attributed by the addition of trimethoprim.

### Identification of Antimicrobial Resistance Genes

Resistance genes were deducted from whole-genome sequencing of randomly selected 64 *E. coli* (Danish) and 34 *S. delphini* isolates originating from Denmark, Spain, Iceland, the Netherlands, and Finland. Briefly, DNA was isolated from culture material using a Maxwell®16 equipment and the 16 LEV Blood DNA Kit according to the manufacturer's instructions (Promega Corporation, USA). The *S. delphini* isolates were treated with lysostaphin before extraction as described in Strube et al. (14). DNA purity and concentration were assessed using NanoDrop ND-1000 (NanoDrop Technologies, USA) and Qubit® (Life Technologies, USA). Library preparation (NextEra XT DNA sample preparation kit, Illumina, USA) and sequencing (Illumina NextSEQ-based technologies in a 150 base pair paired-end configuration with an expected coverage of 50) was outsourced to Statens Serum Institut, Denmark.

Sequences were quality-checked by fastx_quality_stats from the FASTX-Toolkit (FASTX-Toolkit, RRID:SCR_005534) (http://hannonlab.cshl.edu/fastx_toolkit/). Using Centrifuge (Centrifuge Classifier, RRID:SCR_016665), the reads were classified and checked for contamination (15). Contigs were assembled using SPAdes (SPAdes, RRID:SCR_000131) with the setting: settings “-k 21,33,55,77–careful” (16). The assemblies were checked using Quast (QUAST, RRID:SCR_001228) and annotated using Prokka (Prokka, RRID:SCR_014732) (17, 18). Subsequently, antimicrobial resistance genes were identified by running sequences through the ResFinder pipeline (19).

## Results

### *Escherichia coli* 

**Figure 1:**
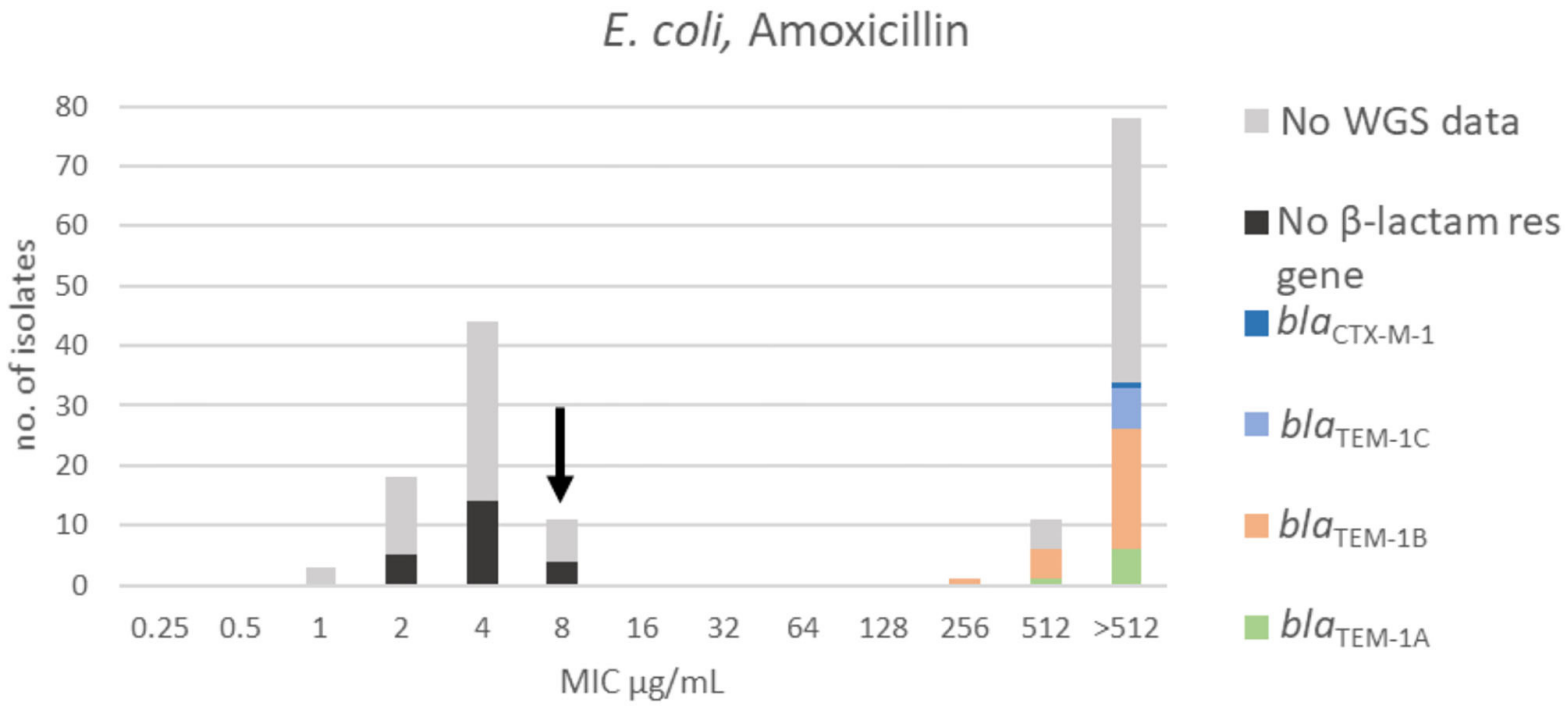
MIC distribution of *E. coli* (*n* = 162) against amoxicillin in the test range of 0.25–512 μg/mL. The arrow indicates the epidemiological cut-off value (ECOFF, EUCAST). Colors indicate if the isolates have been sequenced and whether they harbor known relevant resistance genes. WGS, whole-genome sequencing; res, resistance.

**Figure 2:**
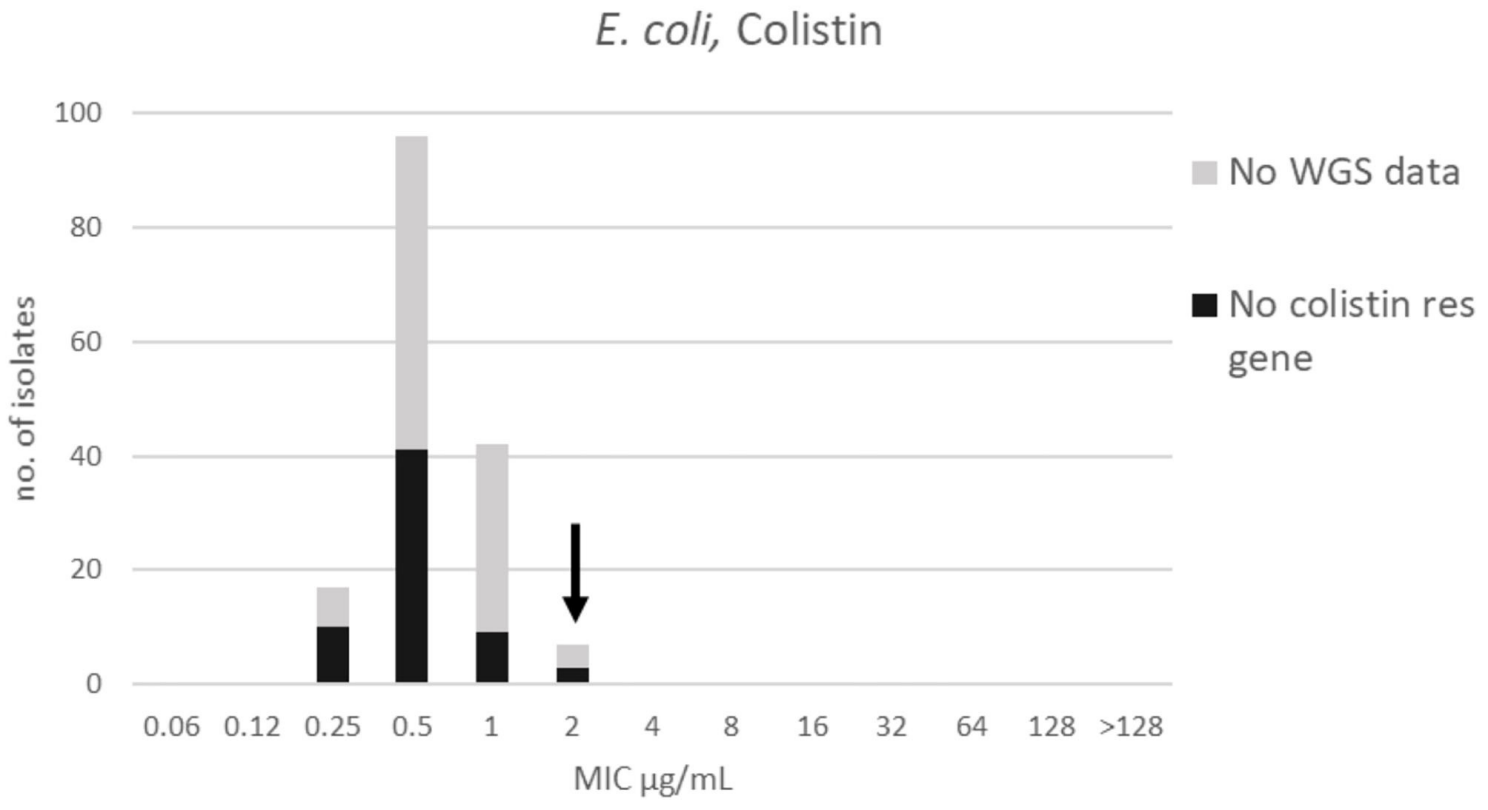
MIC distribution for *E. coli* (*n* = 162) against colistin in the test range 0.06–128 μg/mL. The arrow indicates the epidemiological cut-off value (ECOFF, EUCAST). Colors indicate if the isolates have been sequenced and whether they habor known relevant resistance genes. WGS, whole-genome sequencing; res, resistance.

**Figure 3:**
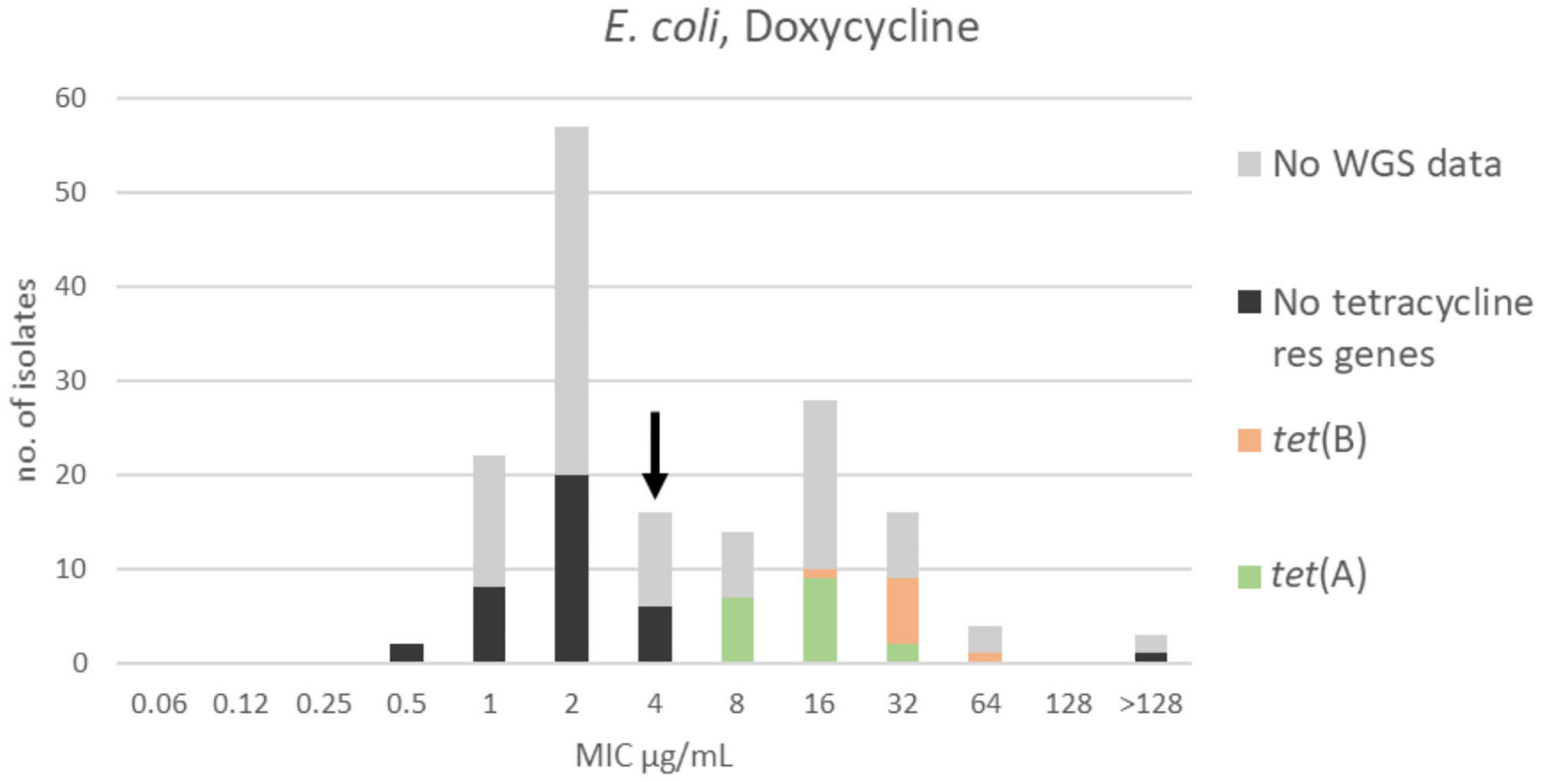
MIC distribution of *E. coli* (*n* = 162) against doxycycline in the test range of 0.06–128 μg/mL. The arrow indicates the epidemiological cut-off value (ECOFF, EUCAST). Colors indicate if the isolates have been sequenced and whether they harbor known relevant resistance genes. WGS, whole-genome sequencing; res, resistance.

**Figure 4:**
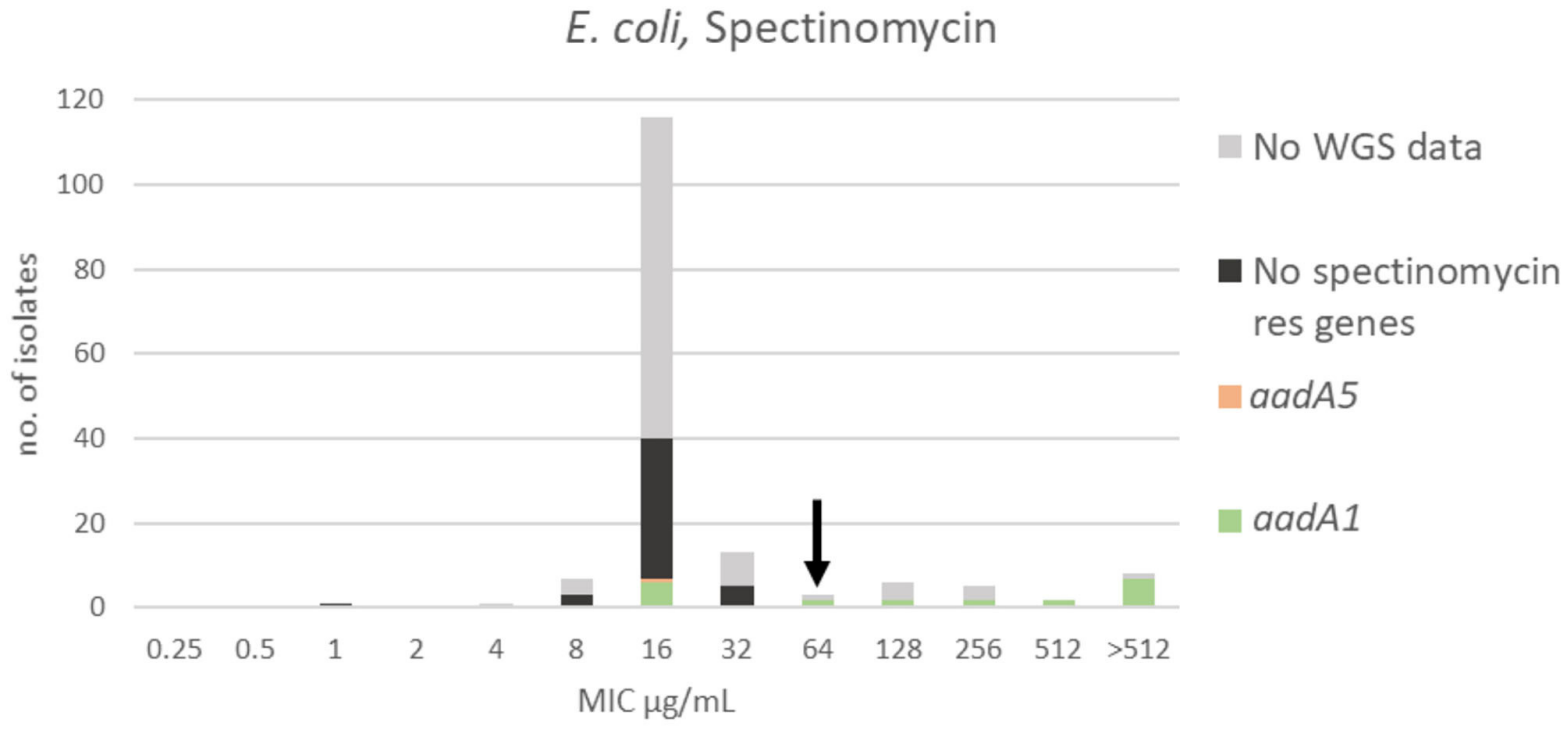
MIC distribution of *E. coli* (*n* = 162) against spectinomycin in the test range of 0.25–512 μg/mL. The arrow indicates the epidemiological cut-off value (ECOFF, EUCAST). Colors indicate if the isolates have been sequenced and whether they harbor known relevant resistance genes. WGS, whole-genome sequencing; res, resistance.

**Figure 5:**
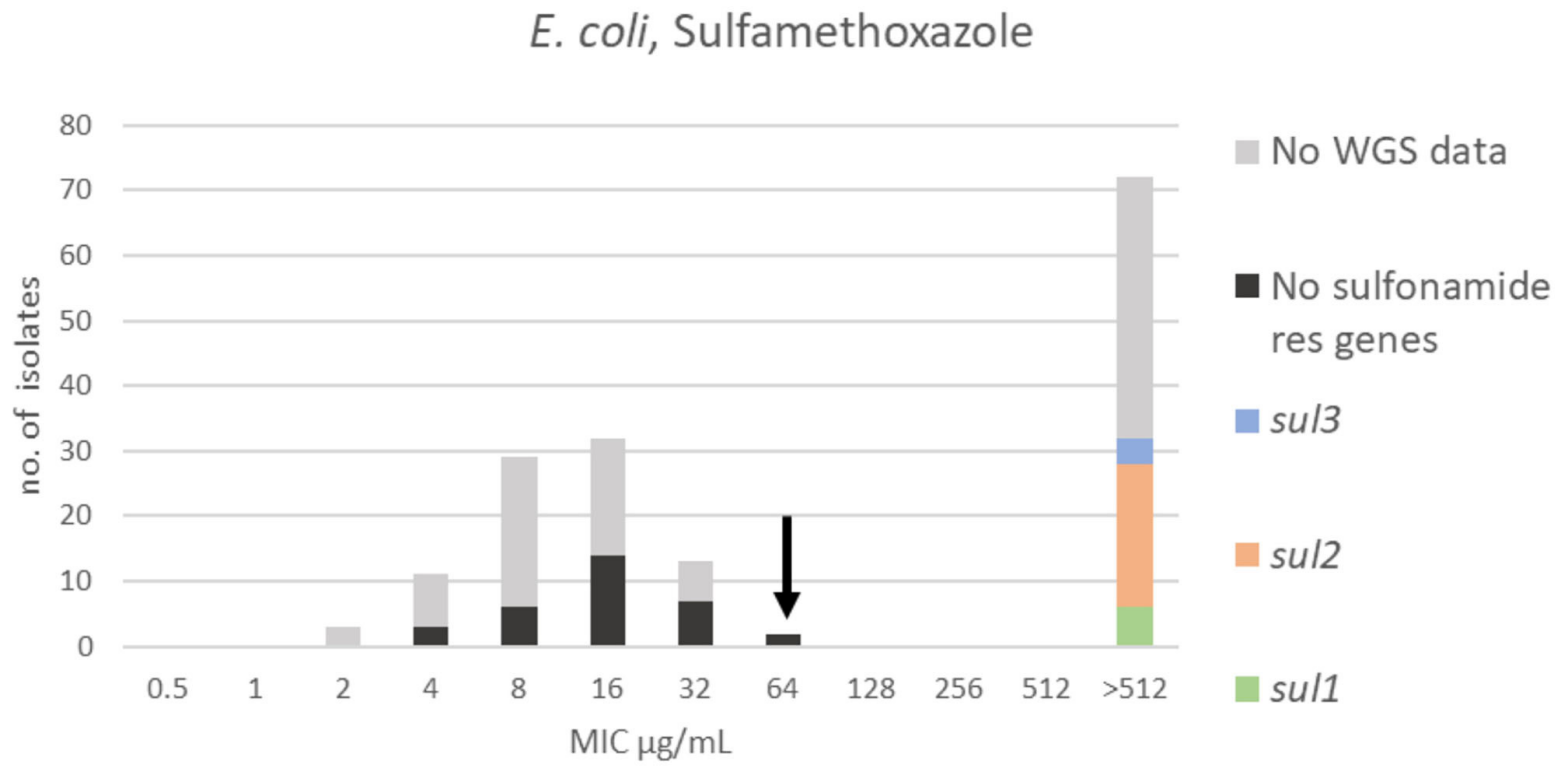
MIC distribution of *E. coli* (*n* = 162) against sulfamethoxazole in the test range of 0.5–512 μg/mL. The arrow indicates the epidemiological cut-off value (ECOFF, EUCAST). Colors indicate if the isolates have been sequenced and whether they harbor known relevant resistance genes. WGS, whole-genome sequencing; res, resistance.

**Figure 6:**
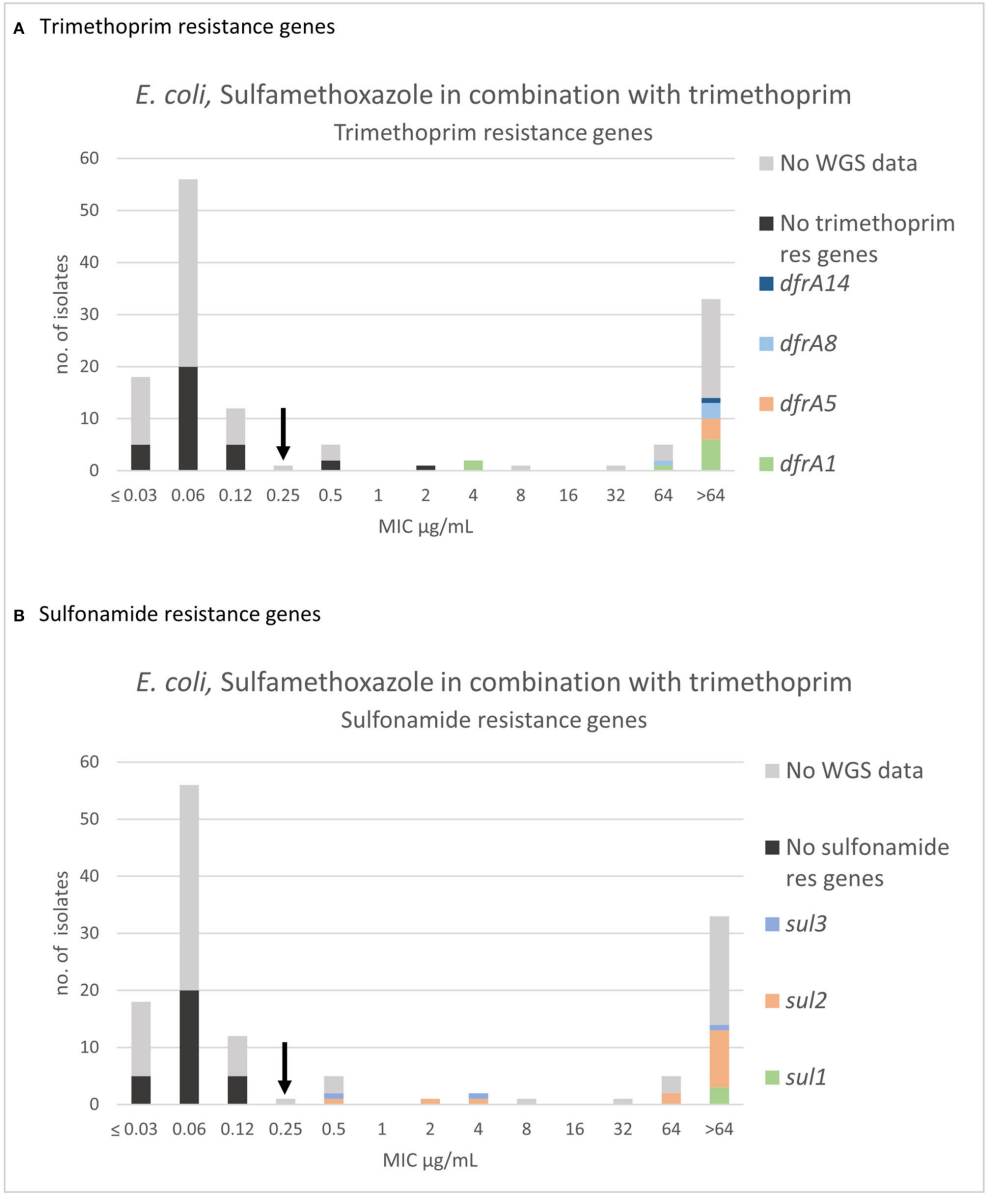
MIC distribution of *E. coli* (*n* = 135) against sulfamethoxazole in combination with trimethoprim (19:1) in the test range of 0.03–64 μg/mL. The arrow indicates the epidemiological cut-off value (ECOFF, EUCAST). Colors indicate if the isolates have been sequenced (*n* = 51) and whether they harbor known **(A)** trimethoprim resistance gene and/or **(B)** sulfonamide resistance genes. WGS, whole-genome sequencing; res, resistance.

For *E. coli*, MIC distributions for six antimicrobial agents are presented (Figures 1–6). Data and derived TECOFFs were in accordance with the EUCAST ECOFFs (Table 2). Antimicrobial resistance genes were not detected in 18 of the 64 sequenced isolates. With only few exceptions, these isolates were found in the wildtype populations (Figures 1–6). With the exception of colistin and spectinomycin, a high number of isolates were part of the non-wildtype populations (Table 6). For three of the agents, the ECOFFinder suggested a value one dilution lower than the EUCAST ECOFF. However, there were no indications other than that the range and mode of colistin, spectinomycin and SXT were in accordance with the EUCAST ECOFFs (Figures 2, 4, 6). Hence, these TECOFFs were visually determined (Table 2).

**Table 2:** *Escherichia coli* isolated from mink—tentative ECOFFs and modes of MIC wildtype distributions and the official ECOFFs from EUCAST.

	**Current study (mink)**		**EUCAST (mixed origins)**	
	**MODE**	**TECOFF**	**MODE**	**ECOFF**
Amoxicillin	4	8	4	8
Colistin	0.5	2^v^	0.5	2
Doxycycline	2	4	2	4
Spectinomycin	16	64^v^	16	64
Sulfamethoxazole	16	64	16	64
Sulfa. + TMP	0.06	0.25^v^	0.06	0.25

*All values are given as μg/mL. The MIC wildtype distributions were visually inspected and the ECOFFs were tested by nonlinear regression analysis using the ECOFFinder 2.0 software (4). Compared with data retrieved from EUCAST (13). ^v^: visually determined, as the MIC distribution was very similar to the EUCAST distribution. Sulfa. + TMP: sulfamethoxazole in combination with trimethoprim (19:1)*.

For amoxicillin, a bimodal distribution was identified. The MIC range, TECOFFand mode for amoxicillin are presented in Figure 1 and Table 2, respectively. Beta-lactam resistance genes were not detected in 23 of the 64 sequenced isolates. All of these were in the wildtype population. Forty-one of the sequenced isolates harbored a β-lactam resistance gene. None of these isolates were in the wildtype population (Figure 1). Genes belonging to the *bla*_TEM−1_ family were most prevalent, while one isolate carried the *bla*_CTX−M−1_ gene encoding an extended-spectrum beta-lactamase (ESBL).

For colistin, the distribution was mono-modal exhibiting a Gaussian distribution in the range 0.25–2 μg/mL (Figure 2). The mode and TECOFF of the colistin MIC values are presented in Table 2. No colistin resistance genes were detected in any of the sequenced *E. coli* isolates (Figure 2).

Two apparently overlapping populations were detected for doxycycline. The range, TECOFF and mode of doxycycline MIC distribution are presented in Figure 3 and Table 2, respectively. The finding of two overlapping populations was supported by the results and distribution of the sequencing data (Figure 3). Three isolates had an MIC > 128 μg/mL and might represent a third population. Thirty-six of the sequenced isolates had no tetracycline resistance genes and were part of the wildtype population. One isolate had no known tetracycline resistance genes despite having an MIC >128 μg/mL. Twenty-seven of the isolates harbored a tetracycline resistance gene [*tet*(A) or *tet*(B)]. None of these isolates were in the wildtype population (Figure 3).

For spectinomycin, the MIC distribution, TECOFF and mode is presented in Figure 4 and Table 2, respectively. Forty-two of the sequenced isolates had no spectinomycin resistance genes and were part of the wildtype population. Twenty-two of the sequenced isolates harbored a spectinomycin resistance gene (*aadA5* or *aadA1*). Seven of these had an MIC < ECOFF, and 15 of these had an MIC > ECOFF (Figure 4).

For sulfamethoxazole, a bimodal distribution was identified. The range, TECOFF and mode of sulfamethoxazole MIC values are presented in Figure 5 and Table 2, respectively. Resistance genes were not detected in 32 of the sequenced isolates. All of these were part of the wildtype population. Thirty-two of the sequenced isolates harbored a sulfonamide resistance gene with *sul2* being the most prevalent. None of these isolates were in the wildtype population (Figure 5).

For sulfamethoxazole in combination with trimethoprim (SXT), three populations were apparent, and the wildtype population displayed a Gaussian distribution in the range 0.03–0.25 μg/mL (Figure 6). A “true” SXT wildtype MIC distribution was solely defined on organisms, which were independently wildtype to both agents. Therefore, 26 sulfamethoxazole non-wildtype and concomitantly SXT wildtype were omitted. One isolate in the SXT wildtype population was omitted due to high trimethoprim MIC (64 μg/mL) (Supplementary Table 1A). All included isolates with SXT MICs of 0.12 μg/mL and 0.25 μg/mL were sensitive to trimethoprim alone (MIC ≤1 μg/mL) (Supplementary Table 1A). Thirty-nine of the isolates with an SXT MIC of 0.06 μg/mL were tested and proved sensitive to trimethoprim alone (MIC ≤ 1 μg/mL) (Supplementary Table 1A). The mode and range of the SXT MIC values are presented in Table 2. There was no indication other than that the MIC distribution from the current study was in accordance with the EUCAST database. All isolates without detected sulfonamide nor trimethoprim resistance genes were in the wildtype population. Three non-wildtype isolates (MIC of 0.5 and 2 μg/mL) harbored only a sulfonamide resistance gene (Figure 6B). Eighteen of the sequenced isolates harbored both sulfonamide (*sul1, sul2, sul3*) and trimethoprim resistance genes (*dfrA1, dfrA5, dfrA8, dfrA14*). Of these 18 isolates, 14 had an MIC > 64 μg/mL, two had an MIC = 64 μg/mL, and two had an MIC = 4 μg/mL (Figure 6).

### *Staphylococcus delphini* 

**Figure 7:**
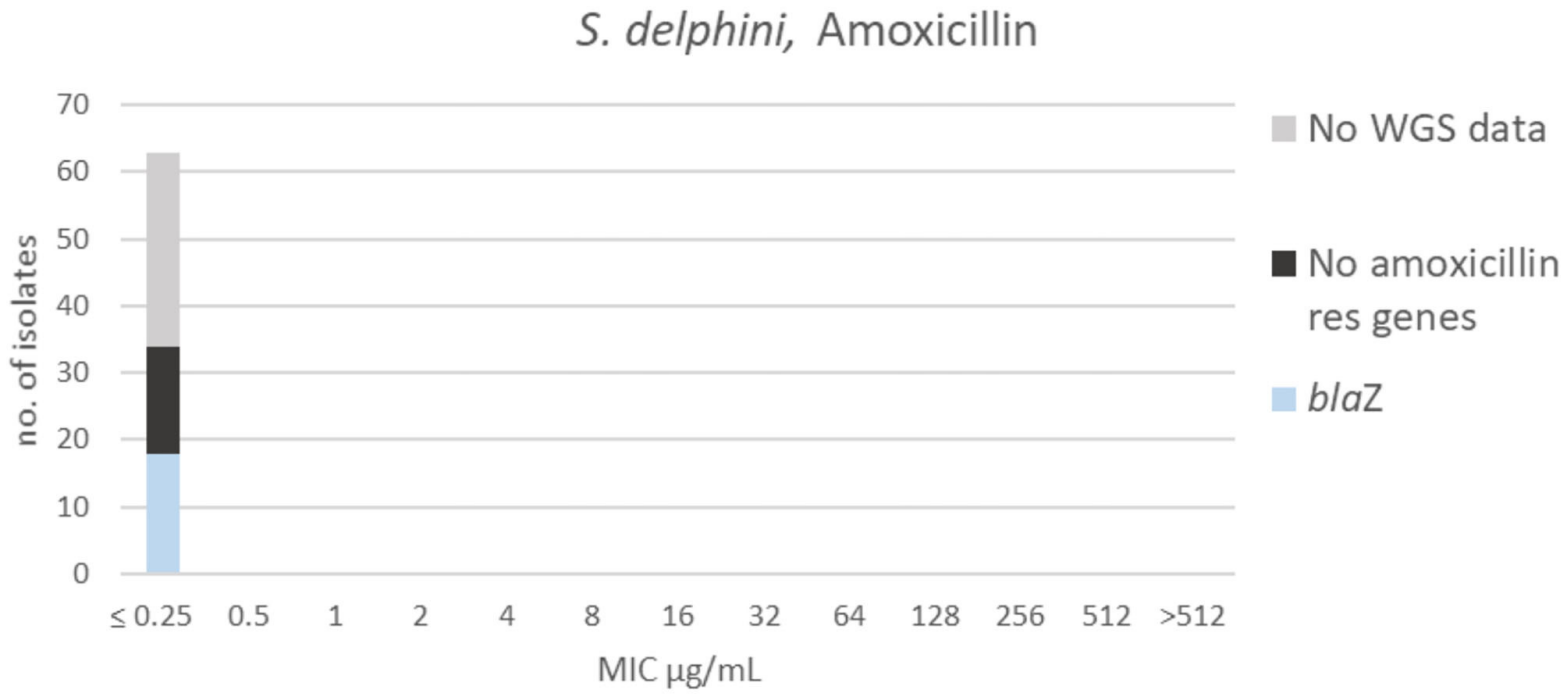
MIC distribution of *Staphylococcus delphini* (*n* = 63) against amoxicillin in the test range of 0.25–512 μg/mL. Colors indicate if the isolates have been sequenced and whether they habor known relevant resistance genes. WGS, whole-genome sequencing; res, resistance.

**Figure 8:**
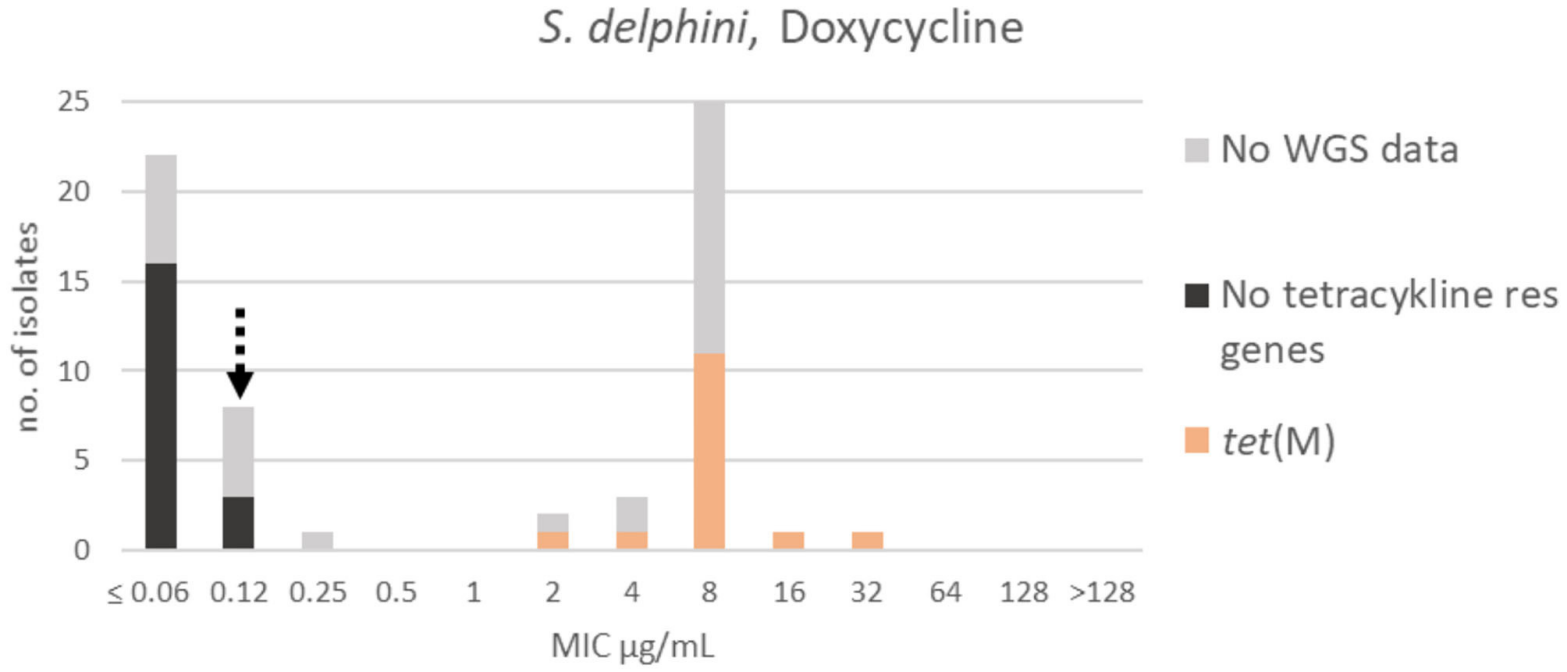
MIC distribution of *Staphylococcus delphini* (*n* = 63) against doxycycline in the test range of 0.06–128 μg/mL. The broken arrow indicates the tentative epidemiological cut-off value (TECOFF). Colors indicate if the isolates have been sequenced and whether they harbor known relevant resistance genes. WGS, whole-genome sequencing; res, resistance.

**Figure 9:**
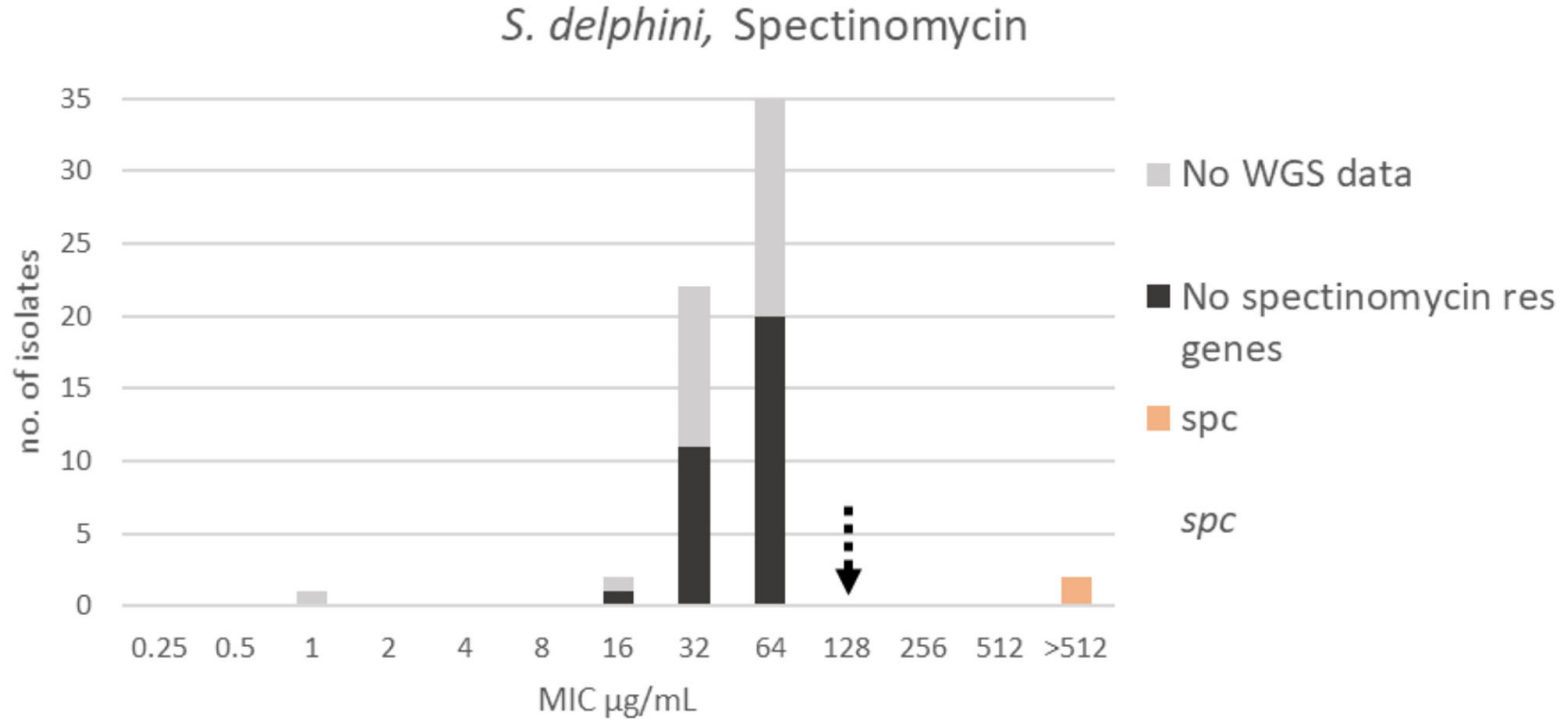
MIC distribution *Staphylococcus delphini* (*n* = 63) against spectinomycin in the test range 0.25–512 μg/mL. The broken arrow indicates the tentative epidemiological cut-off value (TECOFF). Colors indicate if the isolates have been sequenced and whether they habor known relevant resistance genes. WGS, whole-genome sequencing; res, resistance.

**Figure 10:**
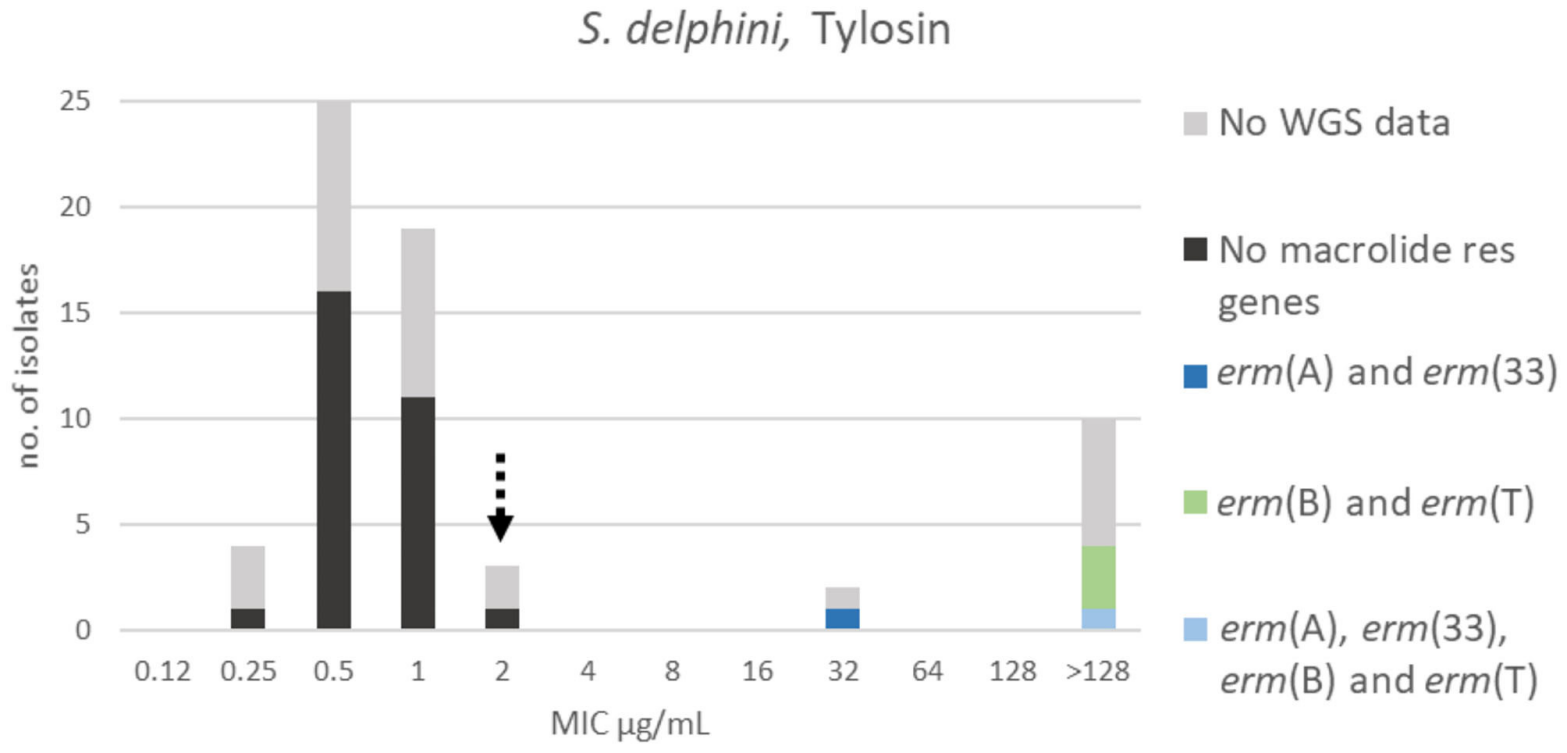
MIC distribution of *Staphylococcus delphini* (*n* = 63) against tylosin in the test range of 0.12–128 μg/mL. The broken arrow indicates the tentative epidemiological cut-off value (TECOFF). Colors indicate if the isolates have been sequenced and whether they harbor known relevant resistance genes. WGS, whole-genome sequencing; res, resistance.

**Figure 11:**
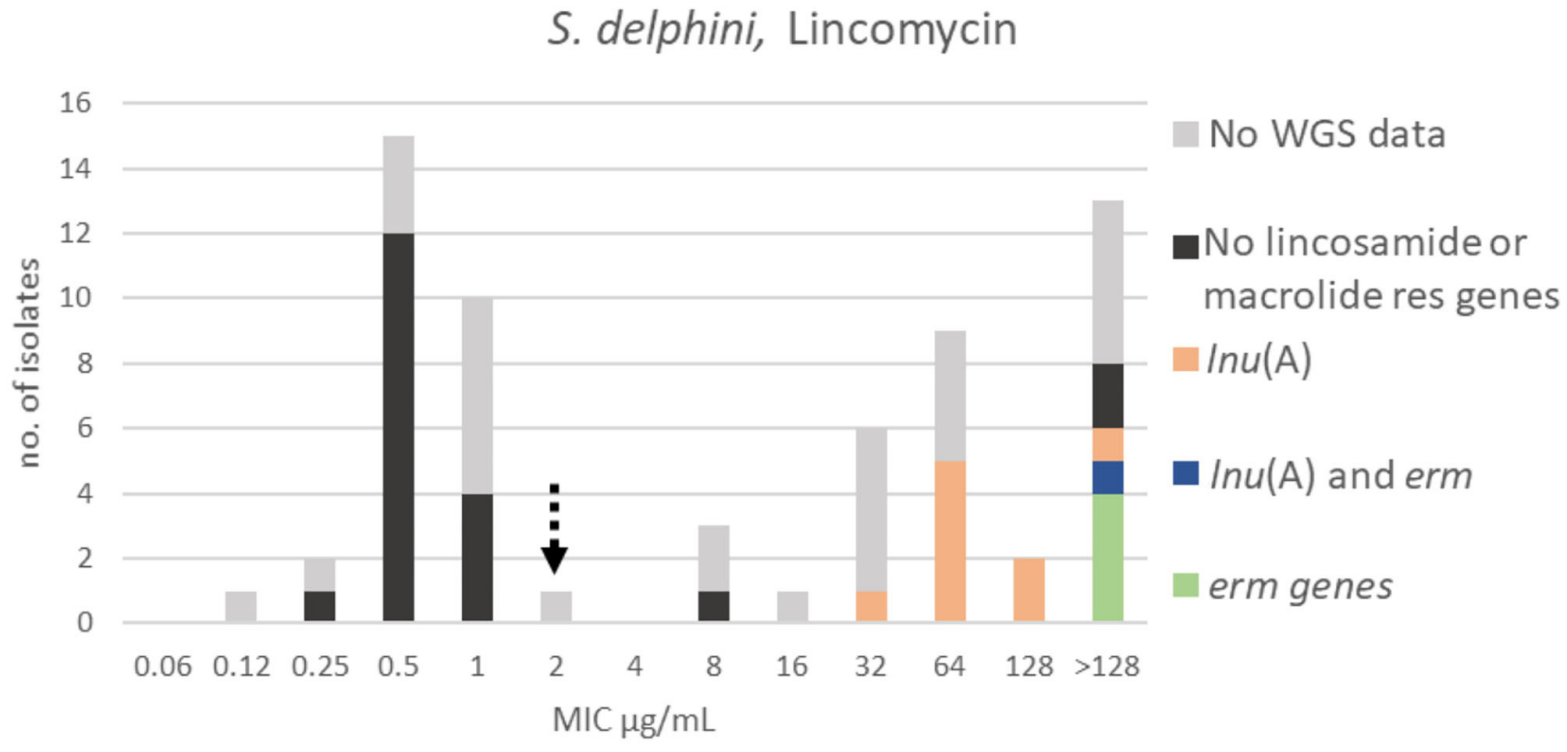
MIC distribution of *Staphylococcus delphini* (*n* = 63) against lincomycin in the test range of 0.06–128 μg/mL. The broken arrow indicates the tentative epidemiological cut-off value (TECOFF). Colors indicate if the isolates have been sequenced and whether they harbor known relevant resistance genes. WGS, whole-genome sequencing; res, resistance.

**Figure 12:**
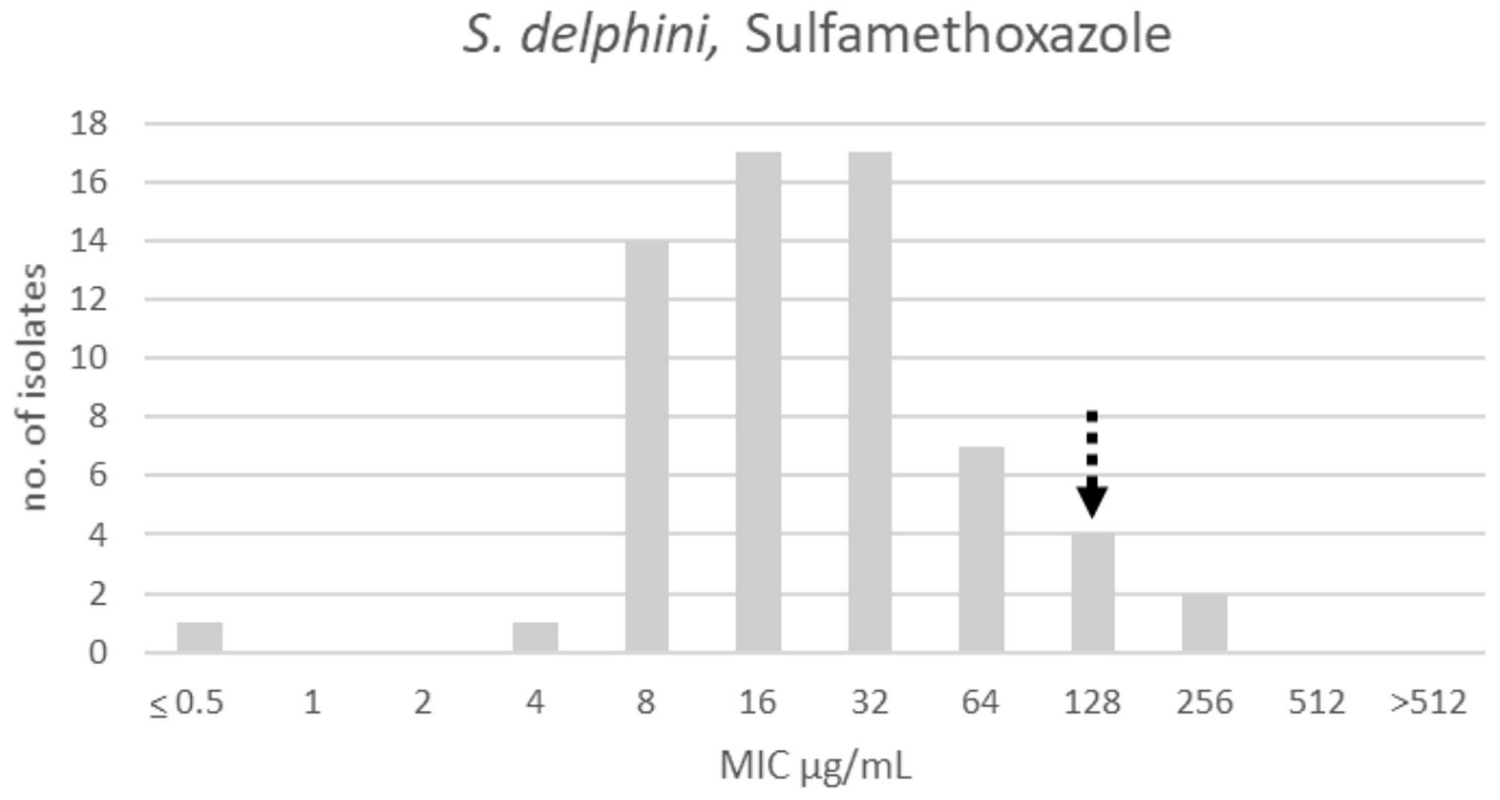
MIC distribution of *Staphylococcus delphini* (*n* = 63) against sulfamethoxazole in the test range of 0.5–512 μg/mL. The broken arrow indicates the tentative epidemiological cut-off value (TECOFF).

**Figure 13:**
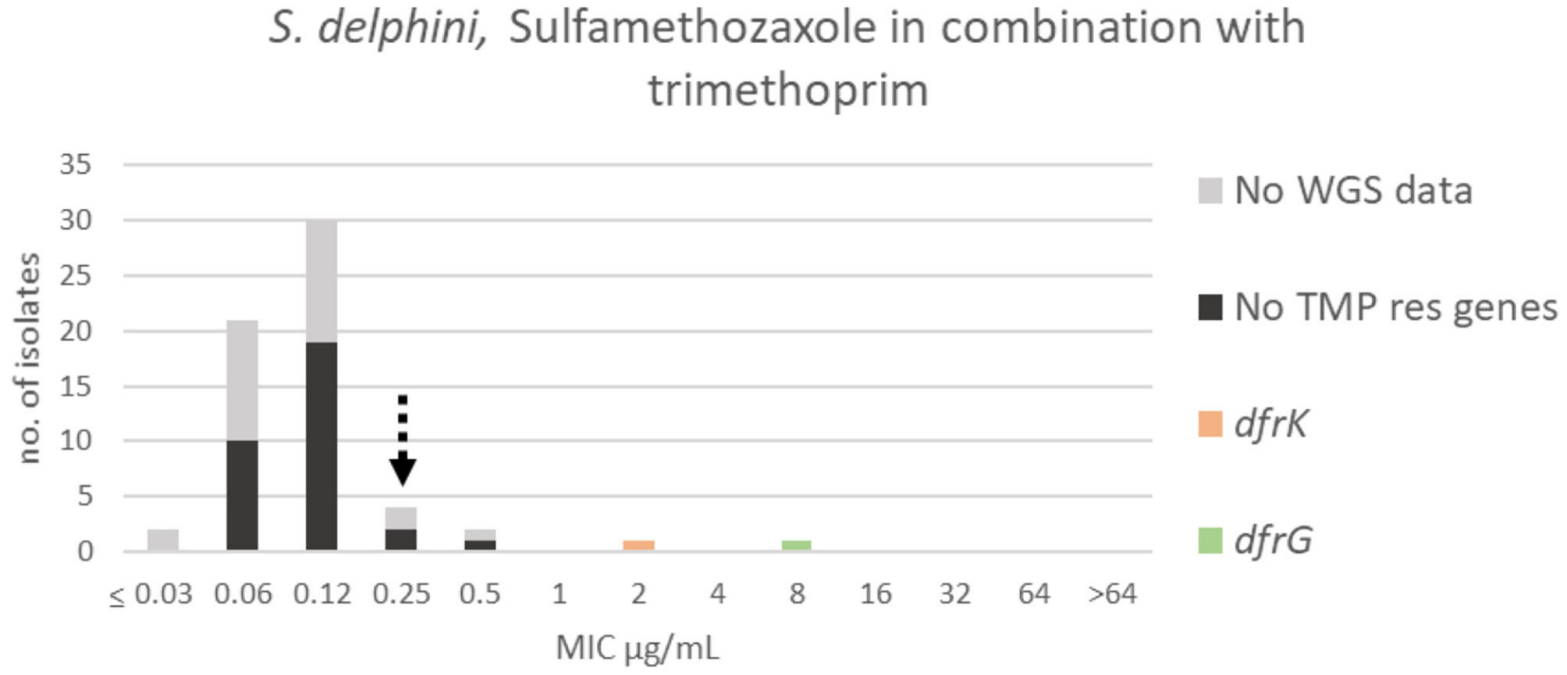
MIC distribution of *Staphylococcus delphini* (*n* = 61) against sulfamethoxazole in combination with trimethoprim (19:1) in the test range of 0.03–64 μg/mL. The broken arrow indicates the tentative epidemiological cut-off value (TECOFF). Colors indicate if the isolates have been sequenced and whether they harbor known relevant resistance genes. WGS, whole-genome sequencing; res, resistance.

For *S. delphini*, the results of the seven tested antimicrobials are presented in Figures 7–13. Tentative ECOFFs were suggested for six of the antimicrobials (Table 3). In seven of the 34 sequenced isolates, no resistance genes were detected, and these isolates were mostly located in the wildtype population (Figures 7–11, 13). For doxycycline and lincomycin, high fractions of isolates were in the non-wildtype populations (Table 6).

**Table 3:** *Staphylococcus delphini* isolated from mink—tentative ECOFFs and modes of MIC wildtype distributions, compared with modes and ECOFFs for *S. aureus* from EUCAST.

	**Current study (mink)**		**EUCAST**, ***S. aureus*** **(mixed origins)**	
	**MODE**	**TECOFF**	**MODE**	**ECOFF**
Amoxicillin	–	–	–	–
Doxycycline	0.06^t^	0.12^t, v^	0.12	0.5
Spectinomycin	64	128^v^	–	–
Tylosin	0.5	2	–	–
Lincomycin	0.5	2	1	2
Sulfamethoxazole	16	128	16	128
Sulfa. + TMP	0.12	0.25	0.06	0.25

*All values are given as μg/mL. The MIC wildtype distributions were visually inspected and the tentative ECOFFs (TECOFFs) were suggested by nonlinear regression analysis using the ECOFFinder 2.0 software (4). Compared with data for S. aureus retrieved from EUCAST (13). ^t^: truncated data, ^v^: visually determined, Sulfa. + TMP: sulfamethoxazole in combination with trimethoprim (19:1)*.

All isolates had an MIC ≤ 0.25 μg/mL to amoxicillin (Figure 7), truncating the dataset to the left. Since the test range did not cover the MIC distribution, it was not possible to suggest a TECOFF. Beta-lactam resistance genes were not detected in 16 of the sequenced isolates. Eighteen of the sequenced isolates harbored the β-lactam resistance gene *blaZ*; these isolates were tested against penicillin. Five of those were non-wildtype against penicillin (MIC ≥ 0.25 μg/mL, Supplementary Table 2) when using the EUCAST ECOFF for *S. aureus* (13).

A bimodal distribution was apparent for doxycycline; the wildtype population was truncated to the left in the range ≤ 0.06–0.25 μg/mL. A TECOFF of 0.12 μg/mL was suggested (Table 3, Figure 8). Nineteen of the sequenced isolates had no tetracycline resistance genes and were part of the wildtype population. Fifteen of the sequenced isolates harbored the tetracycline resistance gene *tet*(M), none of these isolates were in the wildtype population (Figure 8).

For spectinomycin, the apparent wildtype population was in the range 16–64 μg/mL, but due to the lack of a Gaussian distribution it was not possible to apply the ECOFFinder 2.0 software [(4), Figure 9]. A TECOFF of 128 μg/mL was suggested by visual inspection (Table 3). Thirty-two of the sequenced isolates had no spectinomycin resistance genes and were part of the wildtype population. Two of the sequenced isolates harbored the spectinomycin resistance gene *spc*, and both had MICs above the test range > 512 μg/mL (Figure 9).

For tylosin, three populations could be identified. The wildtype population displayed a Gaussian distribution in the range 0.25–2 μg/mL. A TECOFF of 2 μg/mL was suggested (Table 3, Figure 10). Twenty-nine of the sequenced isolates had no macrolide resistance genes and were part of the wildtype population. Five of the sequenced isolates harbored macrolide resistance genes, none of these isolates were in the wildtype population (Figure 10). Four different macrolide resistance genes were identified, all belonging to the *erm* gene family encoding macrolide, lincosamide and streptogramin B resistance (MLS_B_).

At least two populations were apparent for lincomycin with the wildtype population displaying a Gaussian distribution in the range 0.12–2 μg/mL. A TECOFF of 2 μg/mL was suggested (Table 3, Figure 11). Twenty of the sequenced isolates had no macrolide nor lincomycin resistance genes, all but three were part of the wildtype population. Ten of the sequenced isolates harbored the lincomycin resistance gene, *lnu*(A); none of these isolates were in the wildtype population. Additionally, five of the sequenced isolates harbored *erm* genes, all had a lincomycin MIC above the test range (>128 μg/mL, Figure 11).

There was only one apparent population for sulfamethoxazole, and a TECOFF of 128 μg/mL was suggested (Table 3, Figure 12).

For SXT, the wildtype population displayed a Gaussian distribution in the range ≤0.03–0.5 μg/mL. Two isolates were omitted, so that all isolates within the SXT wildtype population (Figure 13) were sensitive to sulfamethoxazole alone (MIC ≤ 128 μg/mL) (Figure 12). Thirty-eight randomly selected isolates in the SXT wildtype population were tested against trimethoprim alone, and all were sensitive (MIC ≤ 8 μg/mL) (Supplementary Table 1B). A TECOFF of 0.25 μg/mL was suggested for SXT (Table 2, Figure 13). Two isolates harbored two different trimethoprim resistance genes, *dfrK* and *dfrG*, and these displayed MICs of 2 and 8 μg/mL, respectively (Figure 13).

### *Streptococcus canis* 

The MIC distributions of *S. canis* are presented in Supplementary Figure 1. Tentative ECOFFs were suggested for five of the seven antimicrobials tested (Table 4). With the exception of SXT, a high number of isolates were found in the non-wildtype populations (Table 6).

**Table 4:** *Streptococcus canis* isolated from mink—tentative ECOFFs and modes of MIC wildtype distribution, compared with modes and ECOFFs for *S. pyogenes* from EUCAST.

	**Current study (mink)**		**EUCAST**, ***S. pyogenes*** **(mixed origins)**	
	**MODE**	**TECOFF**	**MODE**	**ECOFF**
Amoxicillin	–^t^	–^t^	0.016	0.06
Doxycycline	–	–	0.12	0.5
Spectinomycin	16	32	–	–
Tylosin	0.12^t^	0.25^t, v^	–	–
Lincomycin	0.25	0.5 ^v^	–	–
Sulfamethoxazole	32	128	–	–
Sulfa. + TMP	0.06	0.12	0.12	0.5

*All values are given as μg/mL. The MIC wildtype distributions were visually inspected and the tentative ECOFFs (TECOFFs) were suggested by nonlinear regression analysis using the ECOFFinder 2.0 software (4). Compared with data for S. pyogenes retrieved from EUCAST (13). ^t^: truncated data, ^v^: visually determined, Sulfa. + TMP: sulfamethoxazole in combination with trimethoprim (19:1)*.

For amoxicillin, all isolates had an MIC ≤ 0.25 μg/mL (Supplementary Figure 1A), truncating the dataset to the left. Since the test range did not cover the MIC distribution, it was not possible to suggest a TECOFF.

The majority of the isolates displayed a Gaussian distribution for doxycycline in the range 8–32 μg/mL (Supplementary Figure 1B). However, this distribution was most likely not the wildtype distribution, since two isolates had MIC values of 0.25 and 2 μg/mL, respectively, and since the ECOFF for the closely related species *S. pyogenes* (Table 4) and *S. pneumoniae* is 0.5 μg/mL (13). Consequently, a TECOFF was not proposed.

Two main distributions were apparent for spectinomycin; the wildtype population displayed a Gaussian distribution in the range 8–32 μg/mL (Supplementary Figure 1C). A TECOFF of 32 μg/mL was suggested (Table 4).

Two distributions were apparent for tylosin, and the wildtype population was truncated in the range ≤0.125–0.25 μg/mL (Supplementary Figure 1D). Visual inspection of the truncated data indicated a tylosin TECOFF of 0.25 μg/mL (Table 4).

Similarly, for lincomycin, two populations were apparent with the wildtype population truncated in the range ≤0.06–0.25 μg/mL (Supplementary Figure 1E). Visual inspection of the truncated data indicated a TECOFF of 0.5 μg/mL (Table 4).

For sulfamethoxazole, probably two overlapping populations were apparent in the range 8–>512 μg/mL. The wildtype distribution was most likely in the range 8–128 μg/mL (Supplementary Figure 1F). A TECOFF of 128 μg/mL was suggested (Table 4).

For SXT, the wildtype population displayed a Gaussian distribution in the range ≤0.03–0.12 μg/mL (Supplementary Figure 1G). Eight isolates were omitted, so that all isolates within the SXT wildtype population were sensitive to sulfamethoxazole alone (MIC ≤ 128 μg/mL, Table 4). Further, 26 randomly selected isolates in the SXT wildtype population were tested against trimethoprim alone, and one isolate with MIC ≥ 4 μg/mL was omitted (Supplementary Table 1C). A TECOFF of 0.12 μg/mL was suggested (Table 4).

### *Pseudomonas aeruginosa* 

For *P. aeruginosa*, MIC distributions and results of the tested antimicrobials are presented in Supplementary Figure 2 and Table 5.

**Table 5:** *Pseudomonas aeruginosa* isolated from mink—tentative ECOFFs and modes of MIC wildtype distributions and the official ECOFF from EUCAST.

	**Current study (mink)**		**EUCAST (mixed origins)**	
	**MODE**	**TECOFF**	**MODE**	**ECOFF**
Colistin	2	4	1	4
Sulfa. + TMP	8	32	–	–

*All values are given as μg/mL. The MIC wildtype distributions were visually inspected and the (T)ECOFFs were tested by nonlinear regression analysis using the ECOFFinder 2.0 software (4). Compared with data retrieved from EUCAST (13). Sulfa. + TMP: sulfamethoxazole in combination with trimethoprim (19:1)*.

For colistin, only one population was apparent (Supplementary Figure 2A). The MIC range and mode for colistin were similar to the EUCAST MIC distribution and the ECOFF of 4 μg/mL (Table 5). All isolates were in the wildtype population (Table 6).

**Table 6:** Percentages of isolates in non-wildtype population.

	***E.coli***	***S. delphini***	***S. canis***	***P. aeruginosa***
Amoxicillin	56	–	–	–
Colistin	0	–	–	0
Doxycycline	40	52	–	–
Spectinomycin	13	3	31	–
Tylosin	–	19	57	–
Lincomycin	–	54	67	–
Sulfamethoxazole	46	3	19	–
Sulfa. + TMP	30	6	0	24

*Tentative epidemiological cut-off values (TECOFFs) from this study were applied (Tables 2–5). Sulfa. + TMP: sulfamethoxazole in combination with trimethoprim (19:1)*.

For SXT, the wildtype population displayed a Gaussian distribution in the range 2–64 μg/mL (Supplementary Figure 2B). A tentative ECOFF of 32 μg/mL was suggested (Table 5). The TECOFF places 24% of the isolates in the non-wildtype population (Table 6).

## Discussion

An ECOFF indicates the cut-off for the sensitive wildtype population, whereas a clinical breakpoint indicates the lowest concentration for which treatment is likely to be successful. Often an ECOFF corresponds to a clinical breakpoint, or the ECOFF is a lower concentration than the clinical breakpoint. In the absence of a clinical breakpoint, the ECOFF may be used to infer susceptibility of a pathogen (5). In that regard, it is worth noticing the high proportion of isolates above the ECOFF in some occasions (Table 6); e.g., for *E. coli*, 56% of the isolates were above the amoxicillin ECOFF, while 40 and 46% were above the ECOFF for doxycycline and sulfamethoxazole, respectively. These findings are in accordance with the clinical resistance results found by Nikolaisen et al. (8), who applied clinical breakpoint adapted from other host species and closely related bacterial species. Further, these authors recorded marked differences in resistance between hemolytic and non-hemolytic *E. coli* isolates, i.e., the proportion of resistant isolates was significantly higher for the hemolytic isolates compared to non-hemolytic ones. For *S. delphini*, 52 and 19% were above the TECOFF for doxycycline and tylosin, respectively, which is almost identical to the proportion of resistant isolates found by Nikolaisen et al. (8) for tetracycline (51%), and erythromycin (20%). Likewise, a similarity was seen for *S. canis* where 57% of the isolates were above the tylosin TECOFF (Table 6), while Nikolaisen et al. (8) found 53% resistant to erythromycin using the adapted clinical breakpoints. Thus, there seems to be a good congruence between the number of isolates above the (T)ECOFFs found in this study compared to our knowledge about clinical resistance for these bacterial species (8). High percentages of isolates above the (T)ECOFF may indicate that the chance of clinical cure is low and the risk of selecting for antimicrobial resistance is high. Accordingly, we recommend susceptibility testing for these antimicrobial/pathogen combinations and using the established (T)ECOFFs as surrogate clinical breakpoints.

The ECOFFs are based on phenotypic antimicrobial resistance patterns. In this study, genotypic data on the presence of antimicrobial resistance genes were included for *E. coli* and *S. delphini* to confirm the phenotypic antimicrobial resistance patterns. Overall, the distributions of genotypes support the interpretation of the distributions and evaluation of the ECOFFs. For example, in most cases antimicrobial resistance genes were detected only in isolates with MICs above the (tentative) ECOFF [*E. coli* 96% (154/161), *S. delphini* 100%, (34/34) Figures 1–13].

All *S. delphini* and *S. canis* isolates had amoxicillin MICs ≤ 0.25 μg/mL (Figure 7, Supplementary Figure 1A). However, 18 of the 34 sequenced *S. delphini* isolates harbored *bla*Z. The *bla*Z gene encodes a β-lactamase conferring resistance to certain β-lactam antimicrobials such as penicillins and aminopenicillins but not cephalosporins. Five of these 18 isolates were phenotypically resistant to penicillin with MICs of 0.25 μg/mL (Supplementary Table 2). Other studies have reported isolates being phenotypically sensitive to β-lactam antimicrobials despite harboring *bla*Z (20–23). This can be explained by failure to induce the *bla*Z gene (24) or the use of incorrect penicillin breakpoints (20–23). In that regard, it should be noted that the available penicillin ECOFF for *S. aureus* was applied (13).

The majority of the *S. delphini* isolates were wildtype to tylosin, and all isolates harboring macrolide resistance *erm* genes were above the TECOFF (Figure 10). Some lincosamide and macrolide resistance genes confer cross resistance (MLS_B_) (25). Such cross resistance is visualized in the lincomycin MIC distribution, as the isolates harboring *erm* genes all have lincomycin MICs above the test range (>128 μg/mL, Figure 11). In contrast, *S. delphini* isolates without *erm* genes, but harboring the lincosamide resistance gene *lnu*(A), were only resistant to lincomycin.

The tetracycline resistance genes *tet*(A) and *tet*(B) were identified in all sequenced *E. coli* isolates representing the doxycycline non-wildtype population. However, the two genes allocated differently in the MIC distribution of the non-wildtype population, as *tet*(A) was present in isolates with doxycycline MICs of 8–32 μg/mL, whereas *tet*(B) was found in isolates with slightly higher MICs of 16–64 μg/mL (Figure 3). This difference in doxycycline MIC related to presence of different *tet* genes has been described previously (26). In the doxycycline distribution, three isolates had an MIC that exceeded the test range, > 128 μg/mL (Figure 3). In the EUCAST database, very few *E. coli* with MIC > 64 μg/mL are reported representing only 0.1% of the isolates (13). This proportional difference might indicate that mink have been exposed to a high selection pressure for this drug. One of these mink isolates was sequenced, but interestingly no known tetracycline resistance genes were detected. The mechanism behind the resistance of this isolate is therefore currently unknown.

For the combinational drug SXT, all isolates in the wildtype population were cross-referenced with the results for sulfamethoxazole alone. Isolates with sulfamethoxazole non-wildtype MICs could not truly belong to the wildtype population for the combinational drug and were therefore omitted from the dataset for the combinational drug (*E. coli n* = 26, *S. delphini n* = 2, *S. canis n* = 8). The low MIC values for SXT in these omitted isolates (0.03–0.5 μg/mL) likely reflect an effect of trimethoprim. The majority of the SXT wildtype population was further tested using trimethoprim alone and all except one *S. canis* and one *E. coli* isolate were found to be wildtype with respect to trimethoprim. These two isolates were therefore also omitted from the distribution for the combinatorial drug (Supplementary Tables 1A,C). Hence, the isolates in the SXT wildtype population were all wildtype to sulfamethoxazole alone. Furthermore, all the randomly chosen isolates from the SXT wildtype population that were trimethoprim tested were also wildtype to trimethoprim alone (Supplementary Table 1). The ECOFFs for the individual antimicrobials are of more biological interest than those of the combinational drug, the latter is however more widely applied in veterinary medicine.

*Pseudomonas aeruginosa* displays intrinsic resistance against the majority of the antimicrobials included in this study, except colistin. None of the isolates had a colistin MIC higher than the EUCAST ECOFF (4 μg/mL), so all isolates were wildtype. Colistin is administered orally to mink, but the absorption of colistin from the intestinal tract is known to be minimal (27, 28). Consequently, colistin treatment of the often severe lower respiratory *P. aeruginosa* infection in mink are not feasible. In addition, colistin is categorized as a reserved group of antimicrobials in the WHO's List of Essential medicines (7, 29). Other agents to consider are aminoglycosides and fluoroquinolones, for which intrinsic resistance is not recorded in *P. aeruginosa*. However, aminoglycosides (e.g., neomycin and gentamicin) are also poorly absorbed from the intestinal tract. A systemic effect with high antimicrobial concentration in the lungs would therefore demand each animal to be treated individually by injection, something that is not feasible in modern mink farming. Fluoroquinolones, such as enrofloxacin, can be used orally for systemic infections but are listed as “Highest priority” among critically important antimicrobials (29). These drugs should therefore not be used for treatment of mink, except in particular situations where there are no other alternatives (30). Sulfonamides in combination with trimethoprim are used empirically to treat *P. aeruginosa* mink pneumonia, even though this pathogen is intrinsically resistant to these combinational drugs. Due to the widespread use and allegedly good clinical effect (Tina Struve, Personal communication, February 10, 2020), we have included data for SXT against *P. aeruginosa* (Supplementary Figure 2B). Based on the MIC distribution and the TECOFF, most (76%) mink *P. aeruginosa* isolates are wildtype, but the TECOFF of 32 μg/mL is high (Supplementary Figure 2B). Furthermore, pharmacokinetic studies conducted by our group (31) indicate that a clinical effect of sulfonamide and trimethoprim against *P. aeruginosa* cannot be expected in mink, even for wildtype isolates.

A careful selection of antimicrobial test ranges was done to confirm concordance with a EUCAST ECOFF or to suggest a TECOFF. Despite the wide test ranges, some challenges occurred when interpreting the MIC distribution results; (1) the wildtype population was truncated resulting in the absence of a mode and the ECOFF being impossible to infer, (2) only one distribution was present, in which case, it was most likely the wildtype population, or, (3) the distribution was not truly Gaussian. These problems could be addressed in future studies by increasing the test range further and/or including more isolates.

## Conclusion

With the MIC Sensititre panels, it was possible to verify ECOFFs and determine new TECOFFs for the majority of the tested mink-specific combinations of microorganism and antimicrobial agents. These TECOFFs may serve as surrogate clinical breakpoints when there is reasonable clinical experience with the antimicrobial in mink. Additionally, it can serve as pharmacodynamic data for future determination of dosage regimens and clinical breakpoints. Further MIC and pharmacokinetic studies are needed for most compounds to establish clinical breakpoints for common mink pathogenic bacteria. Results of this study can help as one step to promote prudent use of antimicrobials in mink and decrease the risk of selecting for antimicrobial resistance.

## Data Availability Statement

The whole genome sequence data presented in this study can be found in online repositories. The names of the repository/repositories and accession number(s) can be found below: https://www.ncbi.nlm.nih.gov/, PRJNA613603; https://www.ncbi.nlm.nih.gov/, PRJNA613557. The MIC data are available at https://www.researchgate.net/publication/344441006_TECOFF_data_bacterial_pathogens_from_mink.

## Author Contributions

NN drafted the manuscript. AR, DL, CC, and NN provided raw data. GK, KP, PD, and NN conducted the analysis of the MIC distribution data. ML conducted the analysis of sequence data. MC, TS, LJ, and KP supervised the project. All authors contributed to the article and approved the submitted version.

## Conflict of Interest

The authors declare that the research was conducted in the absence of any commercial or financial relationships that could be construed as a potential conflict of interest.

## Abbreviations

ECOFF: Epidemiological cut-off value
TECOFF: Tentative epidemiological cut-off value
SXT: Sulfamethoxazole in combination with trimethoprim (19:1).
