# Supplementary material for: Employing MIC Data for Mink Pathogens to Propose Tentative Epidemiological Cut-Off Values: A Step Toward Rationalizing Antimicrobial Use in Mink
Source: Front Vet Sci. 2020 Oct 21;7:544594. doi: 10.3389/fvets.2020.544594 (PMC7646457; doi:10.3389/fvets.2020.544594)
Supplement: Supplementary file 1 [file Data_Sheet_1.PDF]

Supplementary Material

1 Supplementary tables

Supplementary table 1: Trimethoprim (TMP) MIC results for A) *Escherichia coli* (n=53), *Staphylococcus delphini* (n=38) and C) *Streptococcus canis* (n=26)

A) *Escherichia coli* (n=53), TMP test range: 1-32 µg/mL, SMX test range: 0.5-512 µg/mL, SXT test range: 0.03-64 µg/mL

Isolate no.	SXT µg/mL	SMX µg/mL	TMP µg/mL
1	0.06	16	1
2	0.06	4	1
3	0.06	8	1
4	0.06	2	1
5	0.06	32	1
6	0.06	8	1
7	0.06	8	1
8	0.06	16	1
9	0.06	16	1
10	0.06	16	1
11	0.06	8	1
12	0.06	16	1
13	0.06	4	1
14	0.06	32	1
15	0.06	4	1
16	0.06	32	1
17	0.06	32	1
18	0.06	8	1
19	0.06	8	1
20	0.06	16	1
21	0.06	16	1
22	0.06	16	1
23	0.06	8	1
24	0.06	16	1
25	0.06	8	1
26	0.06	16	1
27	0.06	8	1

28	0.06	8	1
29	0.06	16	1
30	0.06	32	1
31	0.06	8	1
32	0.06	16	1
33	0.06	16	1
34	0.06	8	1
35	0.06	8	1
36	0.06	32	1
37	0.06	32	1
38	0.06	64	1
39	0.06	32	1
40	0.12	16	1
41	0.12	16	1
42	0.12	16	1
43	0.12	16	1
44	0.12	32	1
45	0.12	16	1
46	0.12	16	1
47	0.12	16	1
48	0.12	16	1
49	0.12	32	1
50	0.12	4	1
51	0.12	32	1
52	0.25	8	1
53	0.25	8	64

B) *Staphylococcus delphini* (n= 38), TMP test range: 0.5-32 µg/mL, SMX test range: 0.5-512 µg/mL, SXT test range: 0.03-64 µg/mL

Isolate no.	SXT µg/mL	SMX µg/mL	TMP µg/mL
1	0.03	0.5	0.5
2	0.06	8	0.5
3	0.06	16	1
4	0.06	32	1
5	0.06	8	2
6	0.06	8	2
7	0.06	8	2
8	0.06	32	2
9	0.06	128	4

10	0.06	16	4
11	0.06	16	4
12	0.06	8	4
13	0.06	4	2
14	0.12	16	1
15	0.12	32	1
16	0.12	16	2
17	0.12	64	2
18	0.12	16	2
19	0.12	32	2
20	0.12	8	2
21	0.12	8	2
22	0.12	16	2
23	0.12	32	2
24	0.12	64	2
25	0.12	64	4
26	0.12	16	4
27	0.12	16	8
28	0.12	32	4
29	0.12	64	2
30	0.12	32	2
31	0.12	16	2
32	0.12	128	4
33	0.12	64	4
34	0.25	128	2
35	0.25	32	2
36	0.25	16	2
37	0.5	64	4
38	0.5	64	1

C) *Streptococcus canis* (n= 26), TMP test range: 0.5-32 µg/mL, SMX test range: 0.5-512 µg/mL, SXT test range: 0.03-64 µg/mL

Isolate no.	SXT µg/mL	SMX µg/mL	TMP µg/mL
1	0.03	16	0.5
2	0.03	64	4
3	0.03	16	0.5
4	0.06	64	0.5
5	0.06	32	0.5
6	0.06	64	0.5
7	0.06	16	0.5
8	0.06	32	0.5

9	0.06	16	0.5
10	0.06	8	0.5
11	0.06	32	0.5
12	0.06	32	0.5
13	0.06	64	0.5
14	0.06	32	0.5
15	0.06	32	0.5
16	0.06	32	0.5
17	0.06	64	2
18	0.06	64	0.5
19	0.06	64	0.5
20	0.06	128	0.5
21	0.06	64	0.5
22	0.06	64	0.5
23	0.06	32	0.5
24	0.06	32	0.5
25	0.12	64	0.5
26	0.12	128	2

SXT: sulfamethoxazole in combination with trimethoprim (19:1), SMX: sulfamethoxazole, TMP: trimethoprim

Supplementary table 2: Penicillin MIC results for *Staphylococcus delphini* isolates with *blaZ* gene (n=18)

Penicillin test range: 0.06-16 µg/mL. Amoxicillin test range: 0.25-512 µg/mL

Isolate no.	Penicillin µg/mL	Amoxicillin µg/mL
1	0.06	0.25
2	0.06	0.25
3	0.06	0.25
4	0.06	0.25
5	0.06	0.25
6	0.06	0.25
7	0.06	0.25
8	0.12	0.25
9	0.12	0.25
10	0.12	0.25
11	0.12	0.25
12	0.12	0.25
13	0.12	0.25
14	0.25	0.25
15	0.25	0.25
16	0.25	0.25

17	0.25	0.25
18	0.5	0.25

2 Supplementary figures

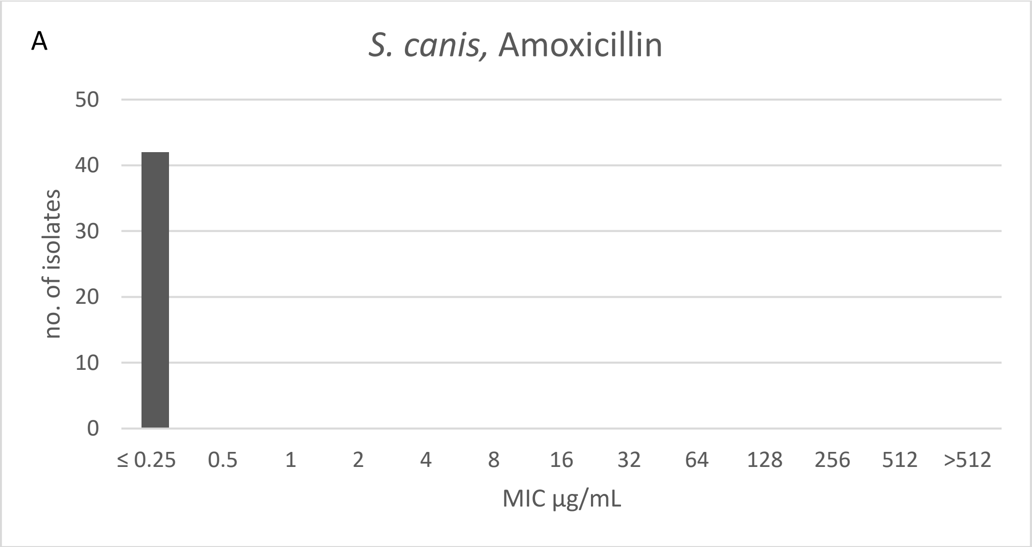

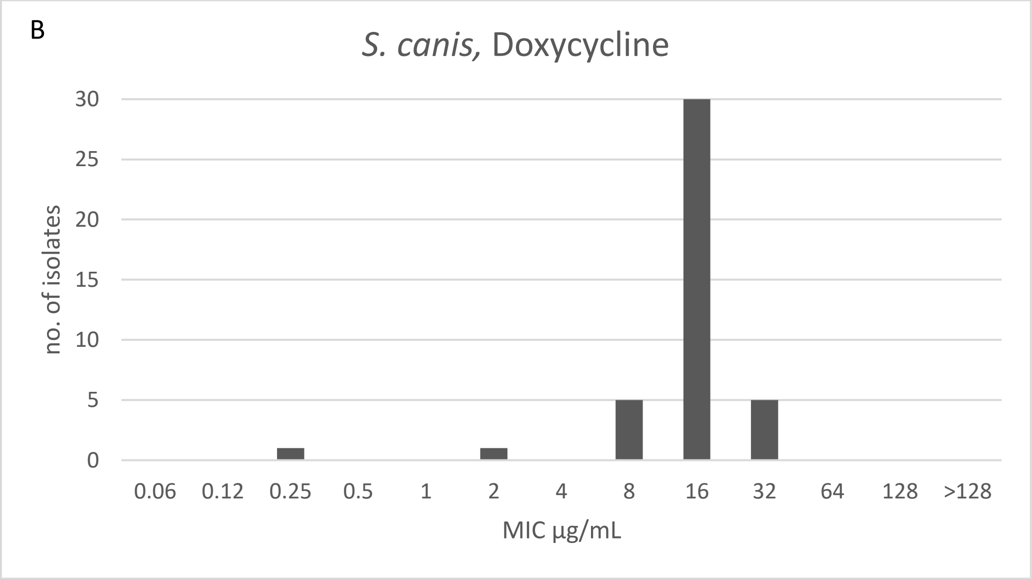

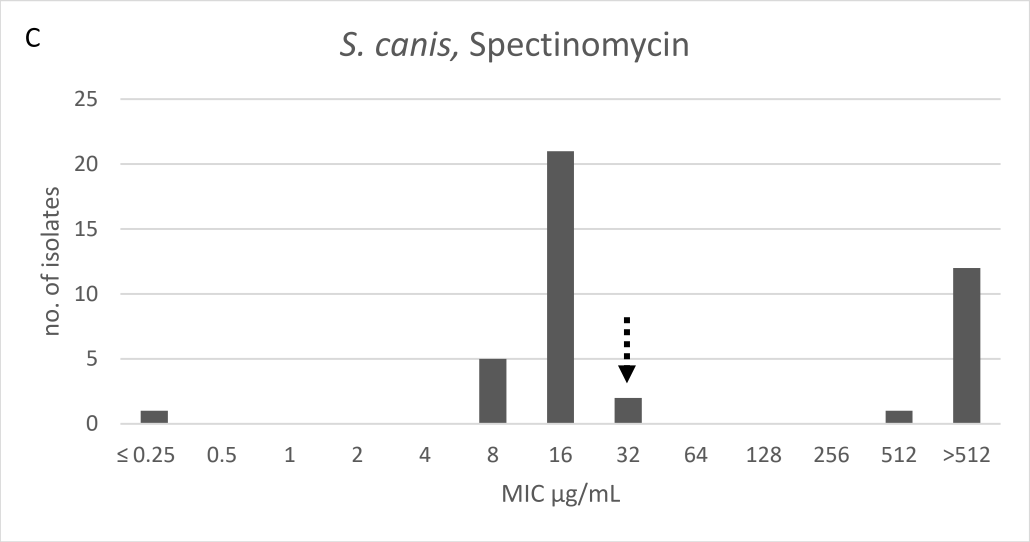
C
S. canis, Spectinomycin
25
20
15
10
5
0
no. of isolates
≤ 0.25
0.5
1
2
4
8
16
32
64
128
256
512
>512
MIC µg/mL

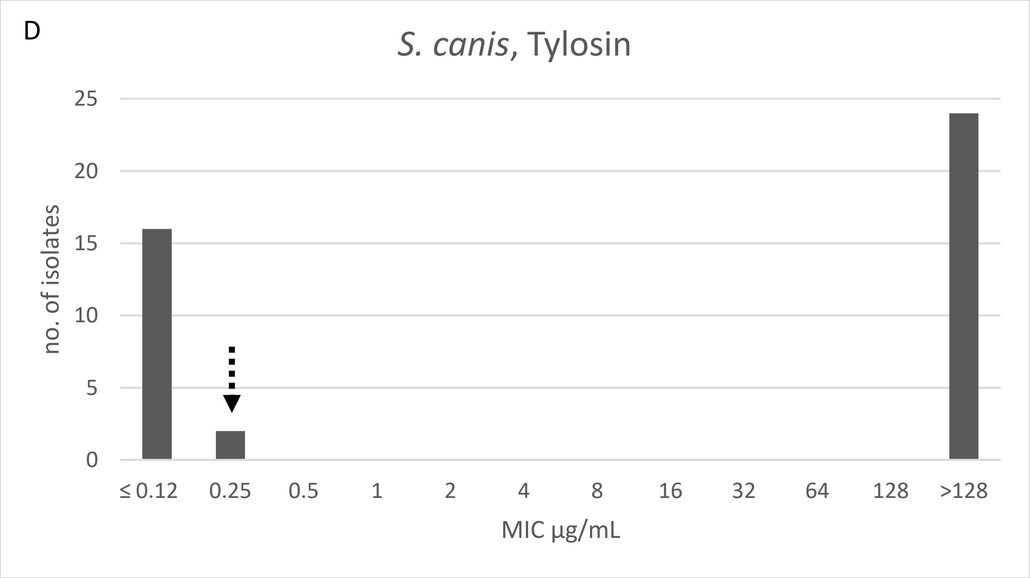
D
S. canis, Tylosin
25
20
15
10
5
0
no. of isolates
≤ 0.12
0.25
0.5
1
2
4
8
16
32
64
128
>128
MIC µg/mL

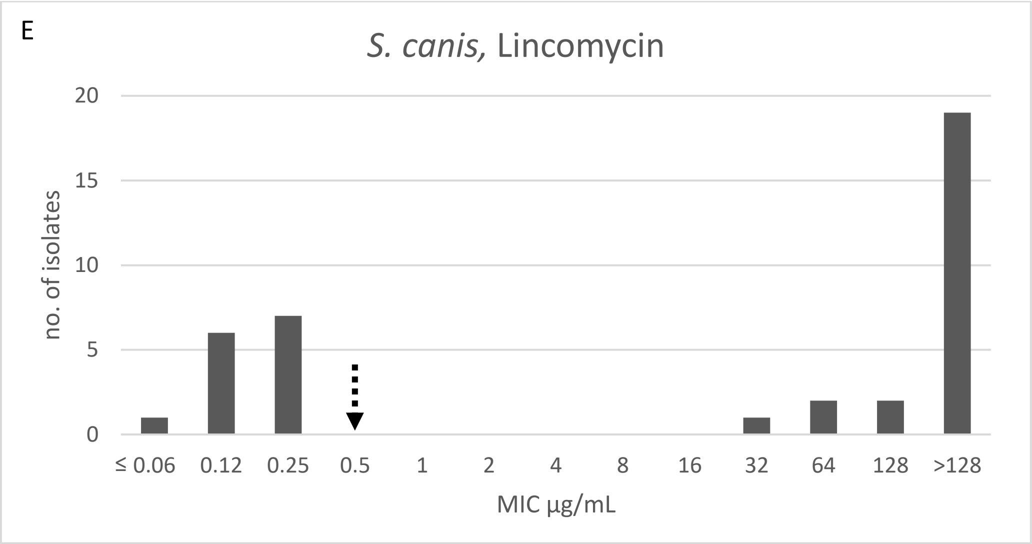
E
S. canis, Lincomycin
20
15
10
5
0
no. of isolates
≤ 0.06
0.12
0.25
0.5
1
2
4
8
16
32
64
128
>128
MIC µg/mL

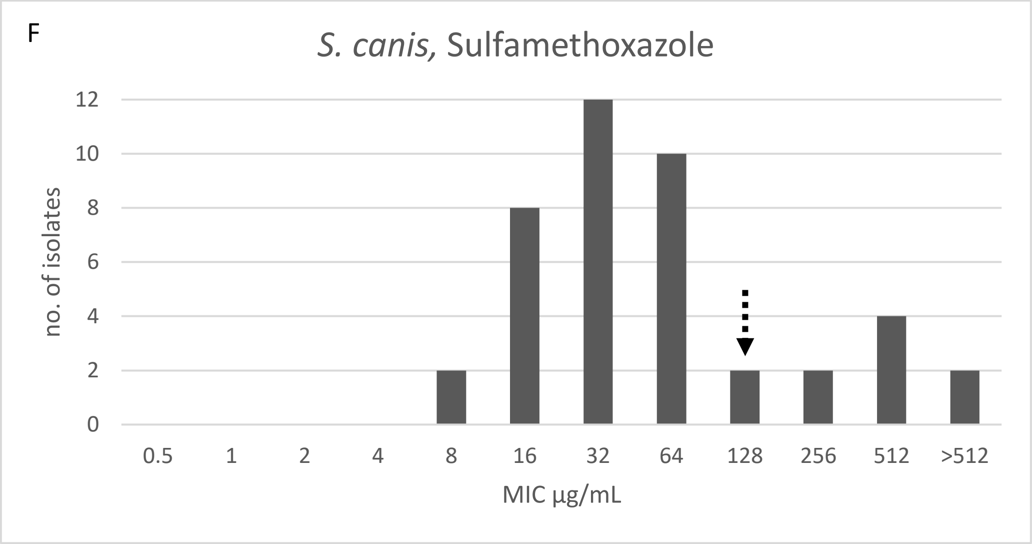
F
S. canis, Sulfamethoxazole
12
10
8
6
4
2
0
no. of isolates
0.5
1
2
4
8
16
32
64
128
256
512
>512
MIC µg/mL

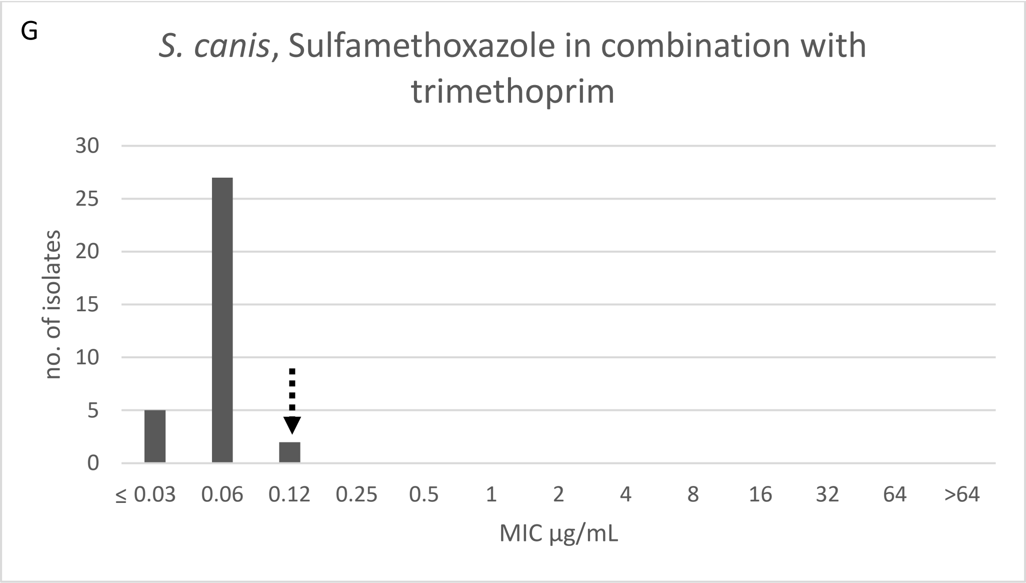

Supplementary figure 1. MIC distribution for *Streptococcus canis* (n=42) against A) amoxicillin, B) doxycycline, C) spectinomycin, D) tylosin, E) lincomycin, F) sulfamethoxazole and G) sulfamethoxazole in combination with trimethoprim (19:1) (n=33). The broken arrow indicates the tentative epidemiological cut-off value (TECOFF).
3G: *S. canis* isolates resistant to either sulfamethoxazole alone (MIC ≥ TECOFF 128 µg/mL) (n=8) or trimethoprim alone (MIC ≥ 4 µg/mL (Nikolaisen et al., 2017)) (n=1) has been omitted. Total number of isolates included in this figure is n=33.

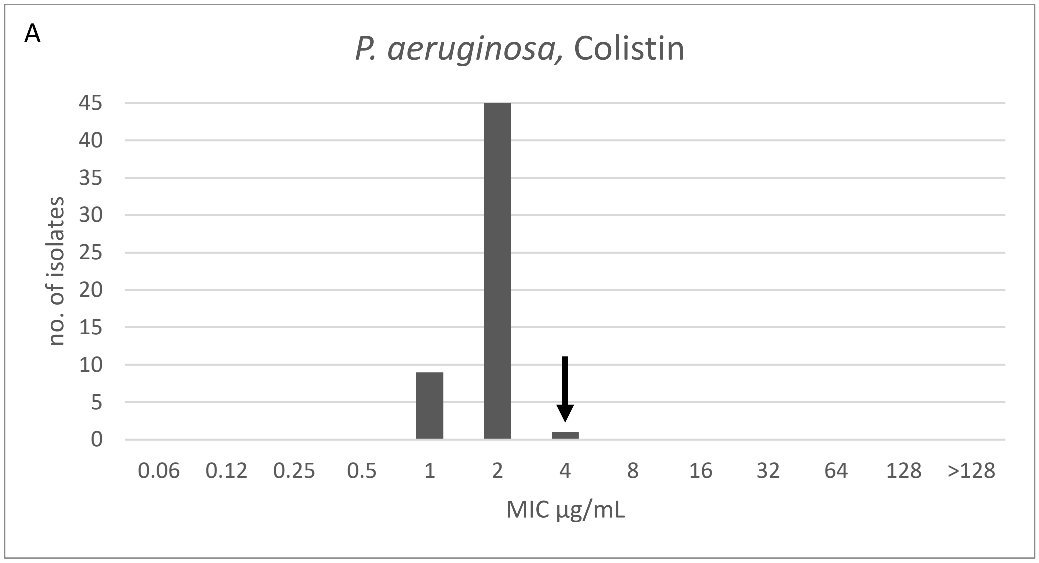

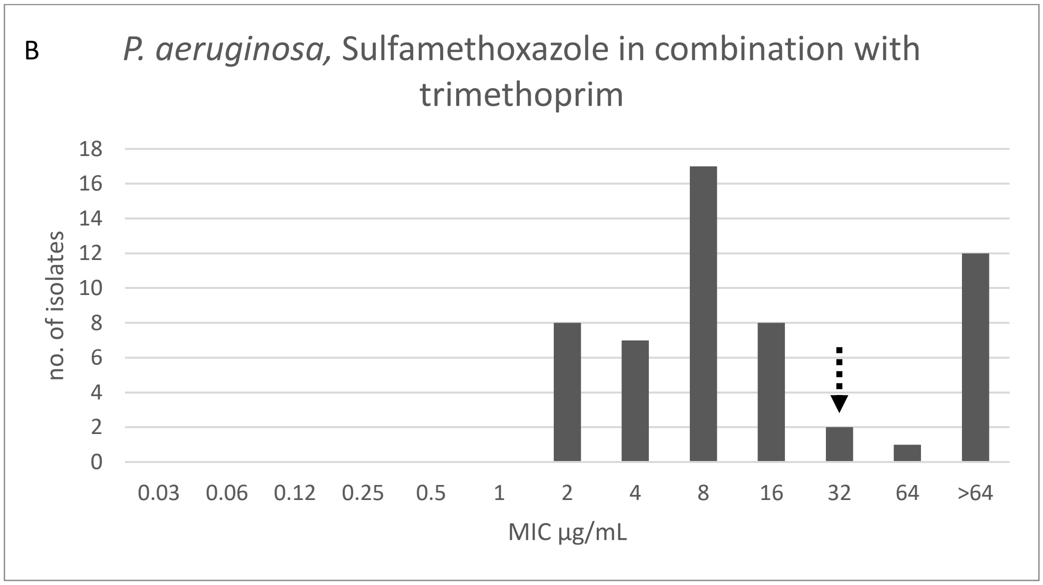

Supplementary figure 2. MIC distribution and (tentative) epidemiological cut-off value (ECOFF = arrow, TECOFF = Broken arrow) for *Pseudomonas aeruginosa* (n=55) against A) colistin and B) sulfamethoxazole in combination with trimethoprim (19:1)
